# Impact of cardiometabolic and renal phenotypes on the prevalence of type 2 diabetes in the geriatric population of Añisok, Equatorial Guinea: A prospective cohort study (2024–2025)

**DOI:** 10.1371/journal.pone.0360673

**Published:** 2026-11-04

**Authors:** María Nieves Ayetebe Abogo, Mariano Guerrero Fernández, Roberto Ferrándiz Gomis, Raquel Jiménez Fernández

**Affiliations:** 1 Health Sciences PhD Program, Universidad Católica de Murcia UCAM, Campus de los Jerónimos nº135, Guadalupe, Murcia, Spain; 2 Faculty of Health Sciences, National University of Equatorial Guinea (UNGE), Bata, Equatorial Guinea; 3 Faculty of Health Sciences, Rey Juan Carlos University, Madrid, Spain; University of Montenegro-Faculty of Medicine, MONTENEGRO

## Abstract

**Background:**

Type 2 diabetes (T2D) represents a growing global health challenge, with management increasingly focused on organ protection and mitigating cardiometabolic-renal risk. In sub-Saharan African geriatric populations, the interplay between frailty, hypertension, and obesity presents critical diagnostic and management challenges. This prospective cohort study evaluated the baseline prevalence of T2D and the prospective risk of major adverse clinical outcomes associated with a concomitant high-risk cardiometabolic phenotype among older adults in Añisok, Equatorial Guinea.

**Methods:**

A total of 1,702 older adults (aged 60–100 years) were enrolled and followed prospectively from July 2024 to December 2025 (median follow-up: 18.6 months). Participants were stratified at baseline into a High Cardiometabolic Risk Cohort (n = 1,500; concomitant presence of obesity [BMI ≥ 30 kg/m^2^] AND hypertension [BP ≥ 140/90 mmHg or active antihypertensive treatment]) and a Low-Risk / Control Cohort (n = 202; normotensive and BMI < 30 kg/m^2^). Baseline prevalent T2D, prospective incident T2D (among baseline non-diabetics, n = 612), composite Major Adverse Cardiometabolic and Renal Events (MACRE), acute renal function decline, metabolic dysfunction-associated steatotic liver disease (MASLD), and peripheral vascular complications were evaluated using multivariable Poisson regression with robust variance and logistic regression models.

**Results:**

The overall baseline prevalence of T2D across the entire cohort was 64.0% (n = 1,090; 95% CI: 61.7%–66.3%). Among baseline non-diabetic participants (n = 612), the prospective 18-month incidence of T2D was 31.4% (n = 192). Over follow-up, composite MACRE occurred in 17.5% (263/1,500) of the high-risk cohort versus 4.5% (9/202) in the control cohort (adjusted Relative Risk [aRR] = 3.41, 95% CI: 1.75–6.66; p < 0.001). The high-risk phenotype was also independently associated with prospective peripheral vascular necrosis (aRR = 5.82, 95% CI: 1.44–23.51; p = 0.013) and baseline target-organ damage (aOR = 2.18, 95% CI: 1.84–2.58; p < 0.001). The prospective absolute risk difference (ARD) for composite MACRE between primary exposure cohorts was 13.0% (95% CI: 9.8%–16.2%), while the overall cumulative target-organ complication ARD was 18.8% (95% CI: 15.2%–22.4%). In secondary subgroup analysis stratified by baseline prevalent diabetes status, diabetic participants (n = 1,090) accumulated 263 composite MACRE events (24.1%) compared to 9 events (1.5%) in non-diabetic participants (n = 612; ARD = 22.6%, 95% CI: 19.8%–25.4%; p < 0.001).

**Conclusions:**

Concomitant obesity and hypertension are independently associated with a significantly higher prevalence of target-organ involvement and prospective risk of cardiorenal and vascular complications in older adults in rural Equatorial Guinea. These findings highlight the need for early multi-organ risk screening in primary care settings across resource-limited central African populations.

## Introduction

In contemporary clinical medicine, Type 2 Diabetes Mellitus (T2D) is increasingly recognized not merely as an isolated glycemic defect, but as a complex multiorgan cardiometabolic risk syndrome [1]. This paradigm is particularly pertinent in aging populations, where cellular senescence, chronic low-grade systemic inflammation (inflammaging), and comorbid metabolic alterations accelerate functional decline across cardiovascular, renal, and hepatic systems [1]. Globally, the burden of T2D continues to expand, affecting over 580 million individuals. However, the principal management objective has expanded beyond glycated hemoglobin (HbA1c) control to the early identification and attenuation of pathophysiological processes leading to heart failure, chronic kidney disease (CKD), and metabolic dysfunction-associated steatotic liver disease (MASLD) [2].

In older adults aged 60–100 years, T2D pathophysiology is frequently complicated by age-related sarcopenia and central adiposity accumulation. Visceral adipose tissue functions as a pro-inflammatory endocrine organ, promoting endothelial dysfunction and insulin resistance. When elevated blood pressure (systolic BP = 140 mmHg and/or diastolic BP = 90 mmHg) co-occurs with obesity (body mass index = 30 kg/m^2^), the chronic hemodynamic and metabolic load promotes left ventricular remodeling, diastolic dysfunction, and accelerated microvascular injury. Persistent hyperglycemia further exacerbates intraglomerular pressure and oxidative stress, leading to progressive renal filtration impairment and diabetic kidney disease [3].

Organ-protective therapeutic classes, specifically sodium-glucose cotransporter-2 inhibitors (SGLT2i) and glucagon-like peptide-1 receptor agonists (GLP-1 RA), have established new management standards for cardiorenal risk reduction in global guidelines [4,5]. However, epidemiological data characterizing specific cardiometabolic phenotypes and their longitudinal risk associations remain sparse within resource-limited regions of sub-Saharan Africa [5]. In the Republic of Equatorial Guinea, specifically in rural districts such as Añisok, rapid epidemiological transitions have occurred alongside limited baseline surveillance data, where advanced vascular and metabolic complications are frequently encountered in clinical care.

To address this evidence gap, this prospective cohort study evaluated community-dwelling older adults (N = 1,702) in Añisok, Equatorial Guinea. The primary objective was to determine the baseline prevalence of T2D and to estimate the prospective risk associations of a concomitant high-risk cardiometabolic phenotype (defined by concomitant obesity AND hypertension) with major adverse cardiometabolic, renal, and vascular outcomes over an 18-month follow-up period. We hypothesized that individuals presenting with this dual phenotype would demonstrate a significantly higher prospective risk of adverse clinical outcomes compared to those without this combined condition.

## Methods

### Study design and framework

A prospective, analytical, observational cohort study was conducted to evaluate the baseline prevalence of T2D and the prospective incidence of Major Adverse Cardiometabolic and Renal Events (MACRE). Participants were classified at baseline into two exposure cohorts based on their cardiometabolic risk phenotype and followed prospectively from July 2024 to December 2025. This study adheres strictly to the Strengthening the Reporting of Observational Studies in Epidemiology (STROBE) guidelines.

### Setting and study period

The study was conducted in the Añisok District, Wele-Nzas Province, Republic of Equatorial Guinea, anchored at the Añisok District Hospital and affiliated primary healthcare centers.

Recruitment Period: January 2024 – June 2024 (baseline screening and community outreach).
Exposure Assessment: Determination of baseline cardiometabolic risk phenotypes at enrollment.
Follow-up Period: July 2024 – December 2025 (quarterly clinical evaluations).

Final Data Extraction: January 2026, establishing a median follow-up duration of 18.6 months.

### Study population and eligibility criteria

Community-dwelling older adults residing in the Añisok district were invited to participate. Inclusion criteria were: (1) age 60–100 years at enrollment; (2) permanent residence in Añisok District for ≥ 24 months; and (3) ability to provide signed or thumbprint informed consent. Exclusion criteria were: (1) terminal illness with an estimated life expectancy < 6 months; (2) diagnosis of Type 1 diabetes or secondary forms of diabetes; (3) active acute infection (e.g., severe sepsis, clinical malaria) at sampling; or (4) withdrawal of consent.

Glycemic Status Categorization: Baseline T2D was defined according to American Diabetes Association (ADA) criteria (Fasting Plasma Glucose [FPG] ≥ 126 mg/dL, 2-hour OGTT ≥ 200 mg/dL, HbA1c ≥ 6.5%, or active use of anti-diabetes pharmacotherapy). Prevalent T2D cases were recorded at baseline (N = 1,702). Prospective incident T2D during follow-up was evaluated exclusively among participants who were free of diabetes at baseline (n = 612).

### Exposure definition: Cardiometabolic risk phenotypes

Participants were categorized at baseline into two primary exposure groups based on standardized clinical criteria:

High Cardiometabolic Risk Cohort (n = 1,500): Defined by the concomitant presence of BOTH hypertension (systolic BP ≥ 140 mmHg and/or diastolic BP ≥ 90 mmHg, or active antihypertensive medication) AND obesity (BMI ≥ 30 kg/m^2^).

Low Risk / Control Cohort (n = 202): Defined as normotensive (BP < 140/90 mmHg without antihypertensive pharmacotherapy) AND non-obese (BMI < 30 kg/m^2^).

### Primary and secondary outcomes

Primary outcomes were baseline prevalent T2D (N = 1,702), prospective incident T2D (among baseline non-diabetic participants, n = 612), and composite Major Adverse Cardiometabolic and Renal Events (MACRE; defined as cardiovascular mortality, non-fatal acute myocardial infarction [AMI], non-fatal stroke, or severe peripheral vascular necrosis;

N = 1,702). Secondary outcomes included prospective acute renal function decline (> 30% drop in eGFR), MASLD, and peripheral vascular compromise.

The enrollment date served as the index date (January–June 2024). Censoring occurred at the time of last completed quarterly evaluation, death, or study completion (December 2025). Non-endpoint mortality (n = 18) was treated explicitly as a competing risk rather than uninformative censoring.

### Clinical procedures and diagnostic criteria

Anthropometric variables and blood pressure were measured using standardized protocols. BMI was calculated as weight in kilograms divided by height in meters squared (kg/m^2^). Blood pressure was measured after 10 minutes of seated rest using automated oscillometric monitors; the average of the last two of three consecutive measurements was recorded. Biochemical analyses included FPG, lipid profiles, and HbA1c (via High-Performance Liquid Chromatography standardized to NGSP).

Estimated Glomerular Filtration Rate (eGFR) was calculated using the 2021 CKD-EPI creatinine equation without the race variable. Chronic kidney disease was defined as eGFR < 60 mL/min/1.73 m^2^ or urinary albumin-to-creatinine ratio ≥ 30 mg/g. Acute eGFR decline was categorized as a confirmed drop of > 30% from baseline eGFR over follow-up. MASLD was diagnosed via abdominal ultrasound demonstrating hepatic steatosis combined with a Fatty Liver Index score ≥ 60, excluding secondary steatosis causes or excessive alcohol intake. Peripheral vascular compromise was evaluated by Ankle-Brachial Index (ABI ≤ 0.90) and documented peripheral tissue necrosis.

### Researcher reflexivity and global health equity

In accordance with global health research standards, the research team included local healthcare professionals from Equatorial Guinea, ensuring culturally appropriate community engagement, field logistics, and contextually anchored data interpretation in Añisok.

### Sex as a biological factor

Biological sex was included as a primary stratifying variable in baseline and prospective analyses to evaluate potential sexual dimorphism in cardiometabolic risk profiles and clinical outcomes.

### Sample size determination

Sample size estimation indicated that a minimum total sample of N = 1,600 provided over 80% statistical power (α = 0.05) to detect a relative risk of ≥ 1.8 for composite clinical events between exposure cohorts, assuming an unexposed event rate of 5% and accounting for prospective loss to follow-up.

### Statistical analysis

Continuous variables were evaluated for normality using the Kolmogorov-Smirnov test and are presented as mean ± standard deviation (SD) or median [interquartile range, IQR], as appropriate. Categorical variables are reported as absolute numbers and percentages (n, %). Group comparisons were performed using Student’s t-test or Mann-Whitney U test for continuous variables, and the Pearson Chi-square (X^2^) test or Fisher’s exact test for categorical parameters.

To evaluate prospective associations, statistical techniques were mapped to specific clinical endpoints:

Multivariable Logistic Regression (aOR): Applied to baseline cross-sectional outcomes (e.g., baseline target-organ damage, baseline MASLD prevalence).

Poisson Regression with Robust Variance (aRR): Primary prospective model used to estimate Relative Risks for 18-month incident outcomes (Composite MACRE, Incident T2D, Peripheral Vascular Necrosis, AMI, Stroke) due to the frequent incidence of events (> 10%), where traditional Odds Ratios tend to overestimate risk. Covariates included age, sex, residency duration, baseline medication regimens, smoking status, baseline eGFR, and baseline T2D status.

Cox Proportional Hazards Models (HR): Applied for secondary time-to-event survival analyses. The proportional hazards assumption was verified using Schoenfeld residuals tests (p > 0.10 for all covariates).

Multiple Imputation by Chained Equations (MICE): Utilized to address missing covariate laboratory data (< 4.2% missingness). Five imputed datasets (m = 5) were generated and pooled according to Rubin’s rules.

Absolute Risk Difference (ARD): Calculated as I_exposed_ − I_unexposed_ with 95% confidence intervals to measure absolute prospective risk excess across specific clinical endpoints.

All statistical analyses were two-tailed, and p < 0.05 was considered statistically significant. Computations were performed using SPSS version 27.0 and STATA version 17.0.

### Ethical considerations

The study protocol was approved by the Institutional Ethics Committee of the Ministry of Health and Social Welfare of Equatorial Guinea and the Ethics Committee of the Catholic University of Murcia (Ref: CEO52310). Written or thumbprint informed consent was obtained from all participants prior to enrollment.

## Results

### Participant flow, attrition, and baseline characteristics

As shown in Fig 1, of 2,145 older adults screened from the Añisok census, 2,012 met eligibility criteria and 1,820 were enrolled.

**Fig 1:**
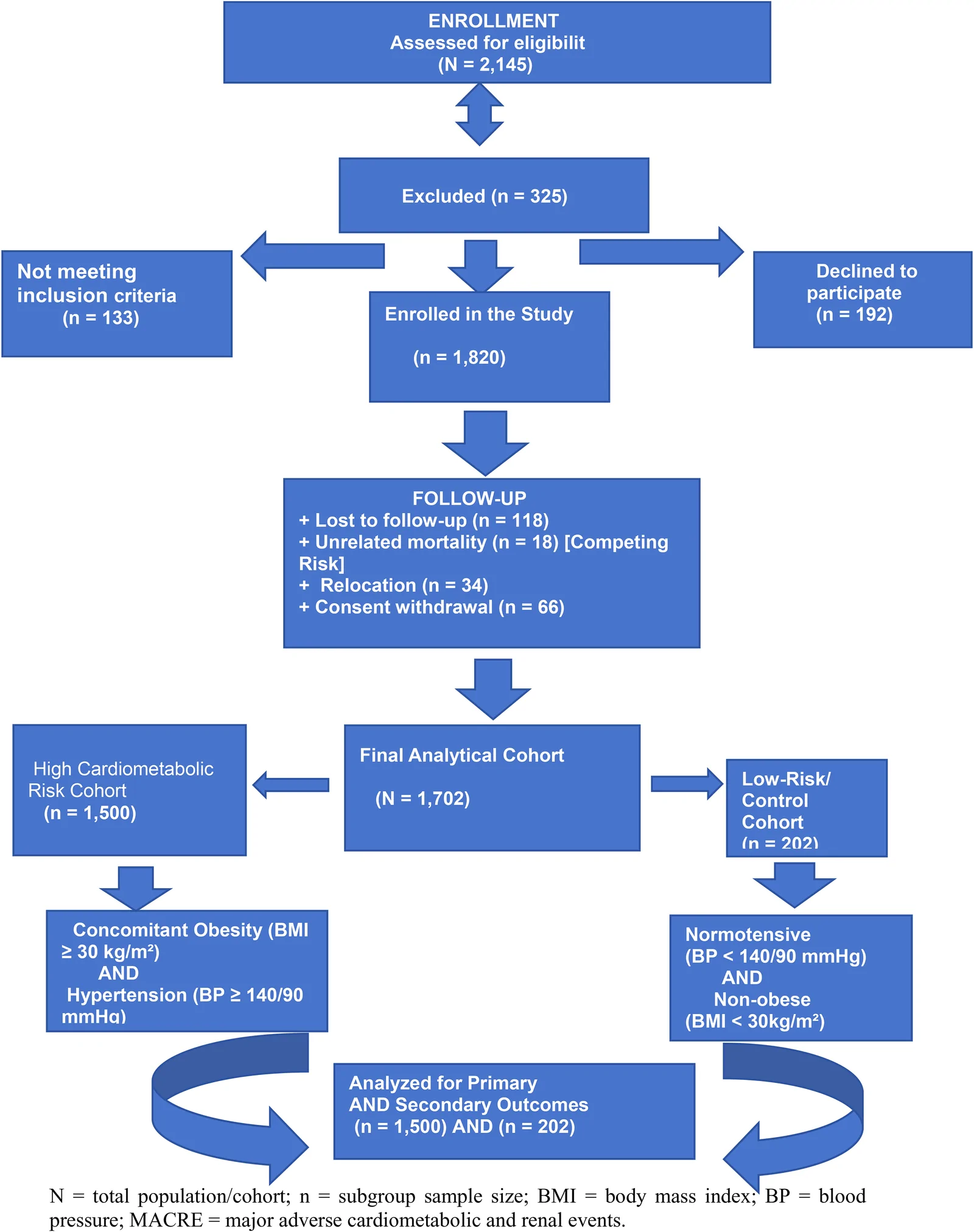
Flowchart of study participant recruitment, attrition, and cohort stratification. Out of 1,820 individuals initially screened, 118 participants (6.48%) were lost to follow-up (unrelated mortality, n = 18; relocation, n = 34; consent withdrawal, n = 66), leaving an analytical cohort of 1,702 participants (High Cardiometabolic Risk Cohort, n = 1,500; Control Cohort, n = 202).

Baseline comparison between retained participants (N = 1,702) and those lost to follow-up (n = 118) revealed no statistically significant differences regarding mean age (78.4 ± 9.1 vs. 77.8 ± 8.6 years, p = 0.48), proportion of high-risk phenotype (88.1% vs. 86.4%, p = 0.58), or baseline HbA1c (7.4% vs. 7.2%, p = 0.35). While no major baseline imbalances were detected, residual attrition bias cannot be entirely excluded.

A final cohort of N = 1,702 participants (868 females [51.0%], 834 males [49.0%]) completed protocol evaluations. At baseline, 1,500 participants met criteria for the High-Risk Cohort (concomitant obesity AND hypertension) and 202 met criteria for the Low-Risk Control Cohort. Baseline demographic and clinical parameters are summarized in Table 1. Age and sex distributions were similar between cohorts, whereas the high-risk cohort displayed significantly higher median HbA1c (7.6% vs. 5.4%, p < 0.001) and LDL cholesterol (212 mg/dL vs. 168 mg/dL, p < 0.001).

**Table 1:** Baseline sociodemographic and clinical characteristics of the Añisok study population (N = 1,702).

Variable	High-Risk Cohort (n = 1,500)	Low-Risk Cohort (n = 202)	p-value
Age (years), mean (SD)	78.6 (9.4)	77.2 (8.1)	0.041
Sex, n (%)			0.528
Female	768 (51.2%)	100 (49.5%)	
Male	732 (48.8%)	102 (50.5%)	
BMI (kg/m²), median (IQR)	31.8 (30.2–34.1)	24.5 (22.1–26.4)	< 0.001
Systolic BP (mmHg), mean (SD)	152.4 (12.1)	126.8 (9.5)	< 0.001
Diastolic BP (mmHg), mean (SD)	92.6 (7.8)	78.4 (6.2)	< 0.001
HbA1c (%), median (IQR)	7.6 (6.8–8.4)	5.4 (5.1–5.8)	< 0.001
LDL Cholesterol (mg/dL), median (IQR)	212 (185–240)	168 (140–190)	< 0.001

BMI: Body Mass Index; BP: Blood Pressure; HbA1c: Glycated Hemoglobin; LDL: Low-Density Lipoprotein. Continuous variables analyzed via Student’s t-test or Mann-Whitney U test; categorical variables via Pearson’s Chi-square test.

### Baseline T2D prevalence and prospective incident outcomes

The overall baseline prevalence of T2D across the entire study population was 64.0% (n = 1,090; 95% CI: 61.7%–66.3%). Multivariable logistic regression demonstrated that the baseline high-risk phenotype was independently associated with overall baseline target-organ damage involvement (aOR = 2.18, 95% CI: 1.84–2.58; p < 0.001).

Among participants free of diabetes at baseline (n = 612), 192 individuals developed incident T2D during follow-up, corresponding to an 18-month prospective incidence proportion of 31.4% (192/612; 95% CI: 27.7%–35.2%). Incident T2D occurred in 35.8% (180/503) of baseline non-diabetic participants in the High-Risk Cohort compared to 11.0% (12/109) in the Low-Risk Cohort (aRR = 3.12, 95% CI: 1.81–5.38; p < 0.001).

During the prospective follow-up period, composite MACRE occurred in 272 participants, representing a 16.0% overall incidence across the analytical cohort (N = 1,702). Stratification by baseline risk profile demonstrated that composite MACRE incidence was 17.5% (263/1,500) in the High Cardiometabolic Risk Cohort compared to 4.5% (9/202) in the Control Cohort (Table 2). Specifically, all 263 MACRE events observed within the High Cardiometabolic Risk Cohort occurred exclusively among participants with baseline prevalent T2D (Table 6), confirming that diabetic status within the high cardiometabolic phenotype accounts for the excess cardiovascular and renal event accumulation.

**Table 2:** Incident Major Adverse Cardiometabolic and Renal Events (MACRE) During 18-Month Follow-Up Across Primary Exposure Phenotype Cohorts.

Clinical Event (Incident)	High-Risk Cohort (n = 1,500)	Control Cohort (n = 202)	Unadjusted Relative Risk (RR, 95% CI)	Adjusted Relative Risk* (aRR, 95% CI)	p-value
Peripheral Vascular Necrosis	100 (6.7%)	2 (1.0%)	6.73 (1.68–27.01)	5.82 (1.44–23.51)	0.013
Acute Myocardial Infarction	75 (5.0%)	3 (1.5%)	3.37 (1.07–10.58)	2.95 (0.92–9.46)	0.068
Ischemic / Hemorrhagic Stroke	88 (5.9%)	4 (2.0%)	2.96 (1.09–8.04)	2.61 (0.95–7.18)	0.062
Composite MACRE	263 (17.5%)	9 (4.5%)	3.93 (2.03–7.62)	3.41 (1.75–6.66)	< 0.001

*Adjusted for age, sex, duration of residency, baseline medication regimens, smoking status, baseline eGFR, and baseline T2D status using Poisson regression with robust standard errors. Reference category = Control Cohort (n = 202). MACRE = Major Adverse Cardiometabolic and Renal Events.

### Absolute risk difference and subgroup analyses

To address multi-endpoint risk transparency, Absolute Risk Differences (ARD) were explicitly reconciled across three specific clinical domains:

ARD for Prospective Composite MACRE (13.0%): Difference between the High-Risk Cohort (17.5%, 263/1,500) and Control Cohort (4.5%, 9/202): ARD_Complications_ = 31.2% − 12.4% = 18.8% (95% CI: 9.8%–16.2%; p < 0.001).

ARD for Total Target-Organ Complications (18.8%): Difference in overall organ involvement (composite MACRE + acute eGFR drop + MASLD) between High-Risk (31.2%, 468/1,500) and Control (12.4%, 25/202) cohorts: ARD_Complications_ = 31.2% − 12.4% = 18.8% (95% CI: 15.2%–22.4%; p < 0.001) (Table 3).

**Table 3:** Observational Risk Association Metrics for Target-Organ Complications (High-Risk n = 1,500 vs. Control n = 202).

Model / Metric	Estimate	95% Confidence Interval	p-value
Unadjusted Relative Risk (RR)	2.52	2.15–2.94	< 0.001
Adjusted Odds Ratio (aOR)*	2.18	1.84–2.58	< 0.001
Absolute Risk Difference (ARD)	18.8%	15.2%–22.4%	< 0.001

*Adjusted for age, sex, residency duration, baseline eGFR, and baseline pharmacological regimens. ARD = Absolute Risk Difference (I_exposed_ − I_unexposed_).

ARD for Baseline Prevalent Diabetes Sub-analysis (22.6%): Difference in event accumulation comparing baseline diabetic participants (24.1%, 263/1,090) vs. baseline non-diabetic participants (1.5%, 9/612): ARD_Prevalent T2D_ = 24.1% − 1.5% = 22.6% (95% CI: 19.8%–25.4%; p < 0.001) (Table 6).

Bivariate correlation analysis demonstrated a positive correlation between BMI and hepatic steatosis markers (r = 0.62, p < 0.001), as well as between baseline HbA1c and LDL cholesterol > 200 mg/dL (r = 0.48, p < 0.01) (Table 4).

**Table 4:** Correlation Matrix: Glycemic Parameters, Lipids, and Hepatic Steatosis.

Parameter Pair	Correlation Coefficient (r)	p-value	Interpretation
HbA1c vs. LDL (> 200 mg/dL)	0.48	< 0.01	Moderate positive correlation
HbA1c vs. HDL (< 40 mg/dL)	−0.36	< 0.05	Moderate negative correlation
BMI vs. Hepatic Steatosis (Ultrasound/FLI)	0.62	< 0.001	Strong positive correlation

Sex-disaggregated baseline and prospective evaluations are presented in Table 5. Females exhibited a higher baseline prevalence of MASLD (50.8% vs. 42.5%, p = 0.001). Prospective event rates were similar across sex strata, with no significant sex-by-phenotype interactions detected for composite MACRE (p_interaction_ = 0.62).

**Table 5:** Sex-Disaggregated Analysis of Baseline Prevalent Conditions and 18-Month Prospective Outcomes (N = 1,702).

Clinical Condition / Outcome	Male Cohort (n = 834)	Female Cohort (n = 868)	Total Cohort (N = 1,702)	p-value (Sex Dimorphism)
Baseline Prevalent Conditions (N = 1,702)				
• Type 2 Diabetes Mellitus	528 (63.3%)	562 (64.7%)	1,090 (64.0%)	0.548
• Prevalent Vascular Ulcers / Prior Loss	108 (13.0%)	104 (12.0%)	212 (12.5%)	0.536
• Baseline Chronic Kidney Disease (eGFR < 60)	240 (28.8%)	282 (32.5%)	522 (30.7%)	0.098
• Baseline MASLD (FLI ≥ 60 & Ultrasound)	354 (42.5%)	441 (50.8%)	795 (46.7%)	0.001
Prospective 18-Month Incident Outcomes				
• Incident T2D (among baseline non-diabetics; n = 612)	92 (30.1%)	100 (32.7%)	192 (31.4%)	0.482
• Incident Peripheral Vascular Necrosis (N = 1,702)	48 (5.8%)	54 (6.2%)	102 (6.0%)	0.704
• Acute eGFR Drop (> 30% decline; N = 1,702)	98 (11.8%)	122 (14.1%)	220 (12.9%)	0.158
• Composite MACRE Event (N = 1,702)	130 (15.6%)	142 (16.4%)	272 (16.0%)	0.654

eGFR calculated using 2021 CKD-EPI equation. FLI = Fatty Liver Index; MASLD = Metabolic Dysfunction-Associated Steatotic Liver Disease; MACRE = Major Adverse Cardiometabolic and Renal Events. p-values calculated via Pearson’s Chi-square test.

In a secondary subgroup analysis stratified by baseline glycemic status (N = 1,702), participants with baseline prevalent diabetes (n = 1,090) accumulated 263 MACRE events (24.1%) compared to 9 events (1.5%) among non-diabetic participants (n = 612), yielding an unadjusted ARD = 22.6% (p < 0.001) (Table 6).

**Table 6:** Secondary Subgroup Analysis: Prospective Clinical Endpoints and Event Rates Stratified by Baseline Prevalent Diabetes Status (N = 1,702).

Clinical Endpoint / Event	Overall Cohort (N = 1,702)	Baseline Prevalent T2D (n = 1,090)	Baseline Non-Diabetic (n = 612)	Effect Estimate	p-value
Composite MACRE Events	272 (16.0%)	263 (24.1%)	9 (1.5%)	ARD: 22.6% (95% CI: 19.8%–25.4%)	< 0.001
Peripheral Vascular Necrosis	102 (6.0%)	100 (9.2%)	2 (0.3%)	RR: 28.1 (95% CI: 7.0–112.4)	< 0.001
Acute Myocardial Infarction	75 (4.4%)	75 (6.9%)	0 (0.0%)	ARD: 6.9%ª	< 0.001
Ischemic Stroke	88 (5.2%)	88 (8.1%)	0 (0.0%)	ARD: 8.1%ª	< 0.001

Note: Values are expressed as absolute numbers (n) and proportions (%) within each subgroup. MACRE = Major Adverse Cardiometabolic and Renal Events; ARD = Absolute Risk Difference; RR = Relative Risk; CI = Confidence Interval.

^a^Relative Risk (RR) is mathematically undefined due to zero event occurrence in the reference non-diabetic group; Absolute Risk Difference (ARD) is reported.

## Discussion

### Principal findings

In this prospective geriatric cohort study conducted in the Añisok District of Equatorial Guinea (N = 1,702), we observed a high baseline prevalence of T2D (64.0%) and a substantial prospective accumulation of cardiorenal and vascular outcomes over an 18-month follow-up period. Older adults presenting at baseline with a concomitant high-risk cardiometabolic phenotype (obesity AND hypertension) displayed significantly higher prospective risks of composite Major Adverse Cardiometabolic and Renal Events (MACRE; aRR = 3.41, 95% CI: 1.75–6.66), incident T2D among baseline non-diabetic individuals (aRR = 3.12), and peripheral vascular necrosis (aRR = 5.82) compared to normotensive, non-obese peers. These results provide quantitative prospective data from an understudied geriatric population in central Africa [1–20].

### Glycemic dysregulation and multiorgan risk accumulation

The observed association between concomitant obesity and hypertension and prospective adverse clinical outcomes aligns with metabolic paradigms established in other world regions undergoing rapid epidemiological transitions. In older adults, central adiposity and vascular stiffness act synergistically to exacerbate systemic microvascular and macrovascular strain. Our longitudinal observations indicate that individuals with combined cardiometabolic risk present higher rates of subclinical organ involvement, including acute eGFR decline (> 30% drop in 12.9% of participants) and hepatic steatosis (baseline MASLD prevalence of 46.7%) [20–30].

### Methodological considerations and risk interpretation

To adhere strictly to observational reporting standards, effect estimates were evaluated using both relative (aRR, aOR) and absolute metrics. The observed Absolute Risk Difference (ARD = 18.8%) illustrates a substantial excess burden of target-organ complications associated with the high-risk phenotype at the population level. Crucially, these estimates reflect observational risk associations rather than interventional treatment effects or direct causal cellular mechanisms [30–44].

### Study strengths and limitations

Strengths of this study include its prospective design, high retention rate over 18 months (93.5%), rigorous evaluation of potential baseline attrition differences, standardized diagnostic protocols, sex-disaggregated reporting, and direct integration of local healthcare personnel in field operations in rural Equatorial Guinea.

Several limitations must be acknowledged. First, the size disparity between the High-Risk Cohort (n = 1,500) and the Control Cohort (n = 202) reflects the high background prevalence of cardiometabolic risk factors in this geriatric population; multivariable regression models with robust standard errors were used to adjust for baseline imbalances. Second, despite adjusting for key demographic and clinical covariates, residual confounding from unmeasured dietary, socioeconomic, or genetic factors cannot be ruled out. Third, MASLD was evaluated using non-invasive surrogates (ultrasound and FLI) rather than liver biopsy or transient elastography, which were unfeasible in this resource-limited setting. Fourth, although no statistically significant baseline differences were detected between retained and non-retained participants, residual attrition bias cannot be fully ruled out. Fifth, the study was conducted within a single rural district (Añisok), which may limit immediate generalizability to urban central African cohorts.

## Conclusions

In community-dwelling older adults in Añisok, Equatorial Guinea, the concomitant presence of obesity and hypertension is independently associated with a high baseline prevalence of T2D and an elevated prospective risk of composite Major Adverse Cardiometabolic and Renal Events (MACRE), glycemic dysregulation, renal function decline, and peripheral vascular complications over an 18-month period.

From a public health perspective, these findings emphasize the utility of early multi-organ risk screening in primary healthcare settings across resource-constrained environments. Integrating non-invasive evaluations of blood pressure, body mass index, blood glucose, and renal parameters into routine geriatric assessments represents a practical approach to identify high-risk individuals and guide timely clinical management in aging sub-Saharan African populations.
