# Supplementary material for: Impact of cardiometabolic and renal phenotypes on the prevalence of type 2 diabetes in the geriatric population of Añisok, Equatorial Guinea: A prospective cohort study (2024–2025)
Source: PLoS One. 2026 Nov 4;21(11):e0360673. doi: 10.1371/journal.pone.0360673 (PMC13734585; doi:10.1371/journal.pone.0360673)
Supplement: Supplementary file 5 [file pone.0360673.s005.docx]

**Assessment of Previous Comments**

The authors have made substantial revisions and have addressed many of the previous reviewer comments. The manuscript is clearer and the authors have provided additional methodological and statistical explanations. However, some important issues remain unresolved and should be corrected before the manuscript can be considered further.

1. **Study population and denominator consistency:** Partially addressed. The cohort sizes are clearly described as 1,500 participants in the high-risk group and 202 in the reference group; however, Table 3 still incorrectly presents the groups as n=1,090 and n=612. This requires correction.
2. **Statistical inconsistencies:** Not fully addressed. The reported p-values for AMI and stroke remain inconsistent with their reported confidence intervals, and the peripheral vascular necrosis p-value is also inconsistent between sections/tables. These numerical discrepancies should be carefully checked and corrected throughout the manuscript.
3. **Absolute risk difference:** Not fully addressed. The manuscript reports an 18.8% absolute risk difference based on different outcome rates in different sections (17.5% vs 4.5% and 31.2% vs 12.4%). The authors should clarify the outcome and denominators used and ensure that the reported values are consistent.
4. **Statistical analysis:** Partially addressed. The methods now provide more detail regarding Poisson regression, logistic regression, Cox regression, and multiple imputation. However, the manuscript should clearly indicate which model was used for each reported outcome and ensure consistency between the Methods, Results, tables, and supplementary analyses.
5. **Outcome definitions:** Partially addressed. The manuscript provides definitions for several outcomes, but the distinction between prevalent baseline conditions, incident outcomes, composite MACRE, and individual organ complications should be made clearer to avoid confusion regarding denominators and interpretation.
6. **Interpretation of findings:** Addressed to some extent. The revised Discussion acknowledges the observational nature of the study and limitations; however, some mechanistic statements remain stronger than can be directly supported by the study design and should be phrased more cautiously.
7. **Loss to follow-up:** Partially addressed. Attrition is reported, but the authors should provide sufficient information on participants lost to follow-up and explain whether attrition could have introduced selection bias.

**Overall assessment**

The authors have **addressed many of the previous comments**, demonstrating substantial revision. Nevertheless, **several significant numerical and denominator inconsistencies remain**, particularly in Tables 2 and 3 and in the reported absolute risk differences. It needs **minor revision**. These should be corrected and cross-checked throughout the manuscript before acceptance.
