# Supplementary material for: Impact of cardiometabolic and renal phenotypes on the prevalence of type 2 diabetes in the geriatric population of Añisok, Equatorial Guinea: A prospective cohort study (2024–2025)
Source: PLoS One. 2026 Nov 4;21(11):e0360673. doi: 10.1371/journal.pone.0360673 (PMC13734585; doi:10.1371/journal.pone.0360673)
Supplement: Supplementary file 1 [file pone.0360673.s001.docx]

**Reviewer Report**

**Manuscript title: “Impact of cardiometabolic and renal phenotypes on the prevalence of type 2 diabetes in the geriatric population of Añisok, Equatorial Guinea: prospective cohort study”**

**Recommendation: Major Revision**

This manuscript addresses an important clinical problem and has potential relevance to diabetes and cardiometabolic disease in older adults. However, several methodological and interpretive issues should be addressed before the manuscript can be considered further.

**Major comments**

1. **Study design and outcome definition need clarification.** The manuscript describes a prospective cohort but refers to T2DM prevalence while the eligibility criteria appear to require confirmed T2DM. The authors should clearly distinguish **prevalent from incident diabetes** and explain baseline diabetes status.
2. **Exposure definition is inconsistent.** The high-risk phenotype is described using both “BMI >30 kg/m² and BP >140/90 mmHg” and “and/or.” This must be standardized throughout the manuscript.
3. **Temporal relationship between exposure and outcomes is unclear.** The authors should clearly distinguish conditions present at baseline from complications occurring during follow-up.
4. **Causal interpretation should be reduced.** Statements suggesting that therapeutic inertia or the cardiometabolic phenotype caused organ damage are not supported by this observational design.
5. **NNH interpretation is inappropriate.** The reported NNH should not be interpreted as the number of patients who would develop preventable complications because of therapeutic inertia. An observational risk difference may be reported, but causal language should be removed.
6. **Renal outcomes require clearer definitions.** “GFR drop >30%” and “acute renal failure” should not be used interchangeably. The authors should specify the eGFR equation, baseline renal status, and criteria for acute versus chronic kidney disease.
7. **Statistical methods require clarification.** The adjustment variables differ between sections. The authors should provide a consistent prespecified adjustment strategy and clearly justify the choice of covariates.

**Minor comments**

- Reconcile participant numbers throughout the manuscript and flow diagram.
- Clarify the primary endpoint and sample-size calculation.
- Define MACRE and its individual components precisely.
- Provide confidence intervals for major effect estimates.
- Consider reducing causal and therapeutic recommendations that are not directly supported by the data.

**Overall assessment**

The study is potentially interesting, but **major clarification of the cohort design, outcome definitions, statistical analysis, and interpretation is required**.

**Recommendation: Major Revision.**
