# Supplementary material for: Impact of cardiometabolic and renal phenotypes on the prevalence of type 2 diabetes in the geriatric population of Añisok, Equatorial Guinea: A prospective cohort study (2024–2025)
Source: PLoS One. 2026 Nov 4;21(11):e0360673. doi: 10.1371/journal.pone.0360673 (PMC13734585; doi:10.1371/journal.pone.0360673)
Supplement: Supplementary file 2 [file pone.0360673.s002.docx]

# **RESPONSE TO REVIEWERS AND EDITORIAL BOARD**

**Manuscript Title:** Prospective Evaluation of Type 2 Diabetes Prevalence, Incident Cardiometabolic and Renal Events, and Target Organ Damage in a Geriatric Cohort from Añisok, Equatorial Guinea

**Manuscript ID:** PONE-D-26-38891

**Journal:** PLOS ONE

**Dear Editorial Office,**

Thank you for your review and guidance regarding our submission. Please find below our point-by-point response to the technical issues raised:

**1. Author Name Consistency in Metadata and Manuscript**

We confirm that "María Nieves Ayetebe Abogo" and "Raquel Jiménez Fernández" are the correct names of the co-authors. The typographical errors present in the submission metadata ("Marla" and "Raque l") have been corrected in the online system to match the primary manuscript document exactly.

**2. Data Availability Statement and Institutional Contact**

In accordance with institutional regulations for doctoral research registered in the Spanish national database (TESEO) and institutional repository policies, the full dataset will be deposited in the UCAM Institutional Repository upon completion of the evaluation process.

To comply with *PLOS ONE* policies regarding persistent data availability, requests for access to the minimal dataset can be directed to the following independent institutional body (non-author contact):

"The official email address of the UCAM Research Ethics Committee (CEI) is **comite_etica@ucam.edu**. The committee is managed through the Vice-Rectorate for Research, whose central contact telephone number is **+34 968 27 88 27**."

**nstitutional Data Access Contact Information**

**Institutional Body:** Research Ethics Committee (*Comité de Ética de la Investigación*), Universidad Católica San Antonio de Murcia (UCAM).

**Institutional Email:** comite_etica@ucam.edu

**Telephone (Vice-Rectorate for Research):** +34 968 27 88 27

**Postal Address:**

Campus de los Jerónimos, s/n 30107 Guadalupe, Murcia (Spain)

**Updated Data Availability Statement:**

*"The minimal dataset required to replicate the study findings will be available upon publication in the UCAM Institutional Repository ([http://repositorio.ucam.edu](http://repositorio.ucam.edu)). Interested researchers may also direct data requests to the Research Ethics Committee of the Universidad Católica de Murcia at comite-etica@ucam.edu]. The authors confirm that they had no special access privileges that others would not have."*

We trust these adjustments satisfy the journal requirements and look forward to proceeding with the editorial process.

## **OVERVIEW AND GENERAL RESPONSE**

We extend our sincere gratitude to the Academic Editor and the Reviewers for their meticulous, constructive, and highly valuable evaluation of our manuscript. We fully acknowledge the concerns raised regarding technical soundness, statistical rigor, data availability, and language clarity.

In response to the "Major Revision" decision, we have performed a comprehensive revision of the manuscript. Specifically, we have:

Standardized and unified all participant counts across the text, tables, and methodological descriptions (N = 1,702 total; n = 1,500 high-risk cohort; n = 202 low-risk control cohort).

Provided explicit operational definitions for major adverse cardiometabolic and renal events (MACRE) and each of its individual components.

Reported 95% Confidence Intervals (95% CIs) for all primary effect estimates in the revised Tables (Tables 2 and 5).

Clarified the observational framework for risk metrics (Number Needed to Harm [NNH] and Absolute Risk Increase [ARI]), explicitly removing causal claims.

Incorporated required PLOS ONE compliance statements, including Data Availability, Researcher Reflexivity, Sex as a Biological Factor, and PPI.

Extensively revised the English terminology, syntax, and scientific style to ensure standard PLOS ONE academic quality.

## **RESPONSE TO STRUCTURED REVIEW EVALUATION CRITERIA**

### **1. Technical Soundness and Support of Conclusions**

**Reviewer #1 Rating:** Partly | **Reviewer #2 Rating:** No

**Authors' Response:**

We have thoroughly revised the study design, population eligibility, and outcome reporting to ensure absolute technical soundness:

**Sample Size and Cohort Balance:** We have clarified that the total analytical cohort consists of **N = 1,702** community-dwelling older adults (aged 60–100 years). The exposure cohort comprises **n = 1,500** high-risk participants (concomitant hypertension and obesity), while the reference control cohort comprises **n = 202** low-risk participants (normotensive and non-obese). We have addressed this imbalance in the Limitations section, noting that it reflects the high background prevalence of cardiometabolic risk in this aging population, and confirmed that multivariable models adjusted for baseline imbalances.

**Exclusion Criteria Refinement:** To eliminate acute confounding, we explicitly added exclusion criteria for active acute infectious diseases at sampling time (e.g., clinical malaria, acute sepsis), terminal illness (<6 months life expectancy), Type 1 diabetes, and withdrawal of consent.

**MACRE Definition:** MACRE was defined as a composite outcome comprising: (1) cardiovascular mortality, (2) non-fatal myocardial infarction, (3) stroke, and (4) severe peripheral vascular events/necrosis.

### **2. Statistical Analysis Rigor**

**Reviewer #1 Rating:** No | **Reviewer #2 Rating:** No

**Authors' Response:**

We have completely overhauled the statistical presentation and methodology to meet the highest empirical standards:

**95% Confidence Intervals:** All adjusted effect estimates (aOR, aRR, aHR) in the revised Tables (Tables 2 and 5) and main text are now strictly accompanied by their corresponding 95% Confidence Intervals (95% CIs) and exact p-values.

**Observational Risk Metrics (ARI / NNH):** We have explicitly clarified in the Methods (Section 10) and Discussion that observational Absolute Risk Increase (ARI) and Number Needed to Harm (NNH) are presented solely as population-level excess risk association metrics, avoiding any therapeutic or causal trial inference.

**Confounder Adjustment & Sensitivity Analysis:** Multivariable models adjusted for age, sex, baseline T2D duration, medication use, smoking status, and baseline eGFR. Missing data were handled using Multiple Imputation by Chained Equations (MICE, m=5). Sensitivity analyses confirmed robustness across complete-case vs. MICE-imputed datasets and after excluding borderline HbA1c cases (6.4%–6.6%).

### **3. Data Availability Policy Compliance**

**Reviewer #1 Rating:** No | **Reviewer #2 Rating:** No

**Authors' Response:**

In accordance with PLOS ONE Data Policy, we have included a fully compliant Data Availability Statement in the revised manuscript:

*"The anonymized dataset underlying the findings, study protocols, and statistical code have been deposited in RIUCAM (Institutional Repository of the Catholic University of Murcia; <http://repositorio.ucam.edu/handle/10952>). Institutional data access requests can be directed to the UCAM Ethics Committee / Data Access Unit (contact: [ceea@ucam.edu](mailto:ceea@ucam.edu))."*

### **4. Presentation, Style, and Standard English Usage**

**Reviewer #1 Rating:** No | **Reviewer #2 Rating:** No

**Authors' Response:**

The entire manuscript has undergone rigorous editorial revision by native English academic editors specializing in clinical epidemiology. Key updates include:

Standardization of nomenclature (e.g., adoption of Metabolic Dysfunction-Associated Steatotic Liver Disease [MASLD] per current international consensus).

Correction of grammatical, typographical, and syntax inconsistencies throughout all sections.

Harmonization of all terminology across the Abstract, Methods, Results (Tables 2 and 5), Discussion, and Conclusions.

## **SUMMARY OF MANUSCRIPT REVISION TRACKING**

| Section | Revision Status | Key Modifications Implemented |
| --- | --- | --- |
| **Abstract** | Unchanged | Verified for numerical consistency (N = 1,702; n = 1,500 vs. n = 202). |
| **Materials & Methods** | Extensively Revised | Unified flow, added infection exclusions, MACRE components, 2021 CKD-EPI formula, MICE, 95% CIs, Ethics (Ref: CEO52310), Reflexivity, Sex, PPI, and Data Availability. |
| **Results (Tables 2 & 5)** | Revised | Updated Tables 2 and 5 to incorporate 95% CIs for all estimates and granular MACRE breakdowns. |
| **Results (Tables 1, 3, 4)** | Unchanged | Confirmed numerical stability (N = 1,702). |
| **Discussion & Conclusions** | Extensively Revised | Reframed non-causal observational language, detailed organ damage mechanisms, addressed sample size limitations, and contextualized public health implications. |

We believe these comprehensive revisions fully address the concerns raised by the Academic Editor and Reviewers, rendering the manuscript technically sound, statistically rigorous, fully compliant, and suitable for publication in *PLOS ONE*.

**RESPONSE TO REVIEWER 1 (METHODS SECTION):**

1. **Study Population & Eligibility:** We thank the reviewer for pointing out this inconsistency. We have revised Section 3 of Methods to clarify that participants were enrolled from the general geriatric community regardless of baseline diabetes status. T2D was evaluated at baseline (prevalence) and during prospective follow-up (incidence).
2. **High-Risk Phenotype Definition:** We have standardized the definition throughout the entire manuscript (Methods Section 4). High Cardiometabolic Risk is strictly defined by the **concomitant presence (AND)** of hypertension (≥ 140/90 mmHg) **AND** obesity (BMI ≥ 30 kg/m^2^).
3. **Renal Outcomes & eGFR:** Section 6 now explicitly details the use of the 2021 CKD-EPI equation and distinguishes acute eGFR decline (>30% drop) from chronic renal impairment.
4. **Statistical Models & Time-to-Event:** Section 10 now fully describes the regression models (specifying reference groups and adjusted covariates: age, sex, duration of T2D, baseline medications, smoking, eGFR) as well as the Kaplan-Meier and Cox Proportional Hazards framework.
5. **Data Availability:** As requested by PLOS ONE, the Data Availability Statement (Section 12) has been updated with a non-author institutional contact point (UCAM Ethics Committee).

**Response regarding Statistical Analysis, Risk Estimates (ARI/NNH), and Causal Interpretation:**

**Point 1: Recalculation and Clarification of ARI and NNH (Reviewer #1 & Reviewer #2)**

**Revision:** We acknowledge the reviewers' critical observation. We have recalculated the absolute risk metrics directly from the reconciled cohort denominators. Furthermore, we have revised all narrative descriptions surrounding NNH.

**Textual Change:** In both the *Methods* and *Discussion* sections, NNH is now defined strictly as an observational metric of excess risk association (NNH= 1 / [Risk High-Risk - Risk Control}). We have removed any wording suggesting that these excess events were "preventable" or "caused by therapeutic inertia."

**Point 2: Clarity of Statistical Models (RR, OR, IRR, and Adjustment Strategy)**

**Revision:** We have expanded Section 10 of the *Methods* to explicitly state:

**Reference Groups:** The Low-Risk cohort (normotensive and non-obese, n = 202) is clearly specified as the reference category across all regression models.

**Model Choice:** Logistic regression was used for cross-sectional binary endpoints (aOR), while Poisson regression with robust standard errors was used for prospective prospective incidence rates (aIRR/aRR).

**Prespecified Covariates:** All adjusted models explicitly incorporate age, sex, T2D duration, baseline medication regimens, smoking status, and baseline eGFR.

**Point 3: Time-to-Event (Kaplan-Meier and Cox Regression)**

**Revision:** We have clarified the time-to-event analytical framework in the *Methods* section. The corresponding Kaplan-Meier curves for composite MACRE survival and Cox Proportional Hazard Ratios (HRs with 95% CIs) are now fully presented in the *Results* section and Figure 2, confirming that proportional hazards assumptions were met (p > 0.05).

**Point 4: Missing Data Procedure (MICE)**

**Revision:** Additional technical detail has been provided for the MICE procedure: m = 5 imputations were generated using predictive mean matching for continuous variables under the MAR assumption, and pooled estimates were derived via Rubin’s rules. A sensitivity analysis comparing complete-case analysis versus MICE-imputed datasets is now presented in Supplementary Table 1.

**Response regarding Numerical Inconsistencies (Tables 2 and 5):**

**Point 1: Numerical Discrepancies in Peripheral Necrosis Data (Reviewer #1 & Reviewer #2)**

**Explanation of Error:** We thank the reviewers for catching this critical error. The discrepancies arose from a mixing of baseline prevalent cases with prospective incident events, alongside a calculation error in the original cross-tabulation of Table 5.

**Correction Applied:**

We have strictly separated baseline **prevalent** complications from prospective **incident** events.

At baseline, there were **212 total prevalent cases** of peripheral vascular necrosis/ulcers

(n = 98 males, n = 114 females).

During the 18-month prospective follow-up, **102 new incident cases** of peripheral necrosis occurred (n = 100 in High-Risk Cohort, n = 2 in Control Cohort; desaggregated as n = 48 males, n = 54 females).

Both Table 2 (incident events) and Table 5 (sex-disaggregated baseline vs. incident data) have been fully reconciled and updated with exact denominators (N = 1,702: n = 1,500 High Risk, n = 202 Control).

**Point 2: Participant Flow Reconciliation**

**Revision:** The numbers across the Methods, Results, Tables 2 and 5, and the newly added **Figure 1 (STROBE Participant Flow Diagram)** now match 100% with no missing or unaccounted participants.

**Response regarding Discussion, Causal Language, and Recommendations:**

**Point 1: Moderation of Causal Language (Reviewer #1 & Reviewer #2)2**

**Revision:** We have thoroughly revised the *Discussion* section to remove any causal assertions regarding "clinical inertia causing events." We now strictly employ observational association terminology (e.g., "strongly associated with," "exhibited a higher cumulative incidence of").

**Textual Refinement:** We explicitly clarified that treatment gaps in Añisok reflect systemic healthcare barriers (e.g., drug supply consistency, diagnostic availability, out-of-pocket expenses) rather than isolated physician failure.

**Point 2: Reinterpretation of NNH**

**Revision:** As detailed in Section 10 of Methods and expanded in the Discussion, NNH is now defined exclusively as an observational metric representing excess population-level risk burden (NNH approx 8 for composite MACRE), avoiding any claim of direct etiology or 100% event preventability.

**Point 3: Pragmatic Framing of SGLT2i and GLP-1RA Recommendations**

**Revision:** We have updated the therapeutic recommendations to be realistic and context-specific for Equatorial Guinea and similar low-resource settings. We propose a tiered implementation framework: first ensuring universal optimization of low-cost essential cardiometabolic drugs (metformin, ACEi/ARBs, statins), followed by targeted, phased introduction of SGLT2 inhibitors for high-risk patients as health system resources allow.
