# Supplementary material for: Impact of cardiometabolic and renal phenotypes on the prevalence of type 2 diabetes in the geriatric population of Añisok, Equatorial Guinea: A prospective cohort study (2024–2025)
Source: PLoS One. 2026 Nov 4;21(11):e0360673. doi: 10.1371/journal.pone.0360673 (PMC13734585; doi:10.1371/journal.pone.0360673)
Supplement: Supplementary file 3 [file pone.0360673.s003.docx]

**Reviewer Comments on the Revised Manuscript**

**Manuscript Title:** *Prospective Evaluation of Type 2 Diabetes Prevalence, Incident Cardiometabolic and Renal Events, and Target Organ Damage in a Geriatric Cohort from Añisok, Equatorial Guinea***.**

**Summary reviewer comments**

**Overall assessment:**
The revised manuscript addresses several of the previous methodological and reporting concerns, including clearer cohort definitions, participant flow, MACRE definition, confidence intervals, missing-data handling, and more cautious causal language. The authors also provide additional information on ethics, data availability, and statistical methods.

However, **several important numerical and methodological inconsistencies remain within the revised manuscript and should be corrected before publication.**

1. **Major inconsistency in T2D prevalence:**
   The Abstract reports an overall T2D prevalence of **52.3% (890/1,702)**, whereas the Results/Table 5 report **64.0% (1,090/1,702)**. These are substantially different figures and cannot both represent the overall baseline prevalence.
   **This should be reconciled throughout the Abstract, Results, Tables, Discussion, and Conclusions.**
2. **Internal inconsistency in outcome data:**
   Different sections report different numbers for MACRE and individual events. For example, one revised Table 2 reports **321 MACRE events**, whereas another Results section reports **272 MACRE events** and Table 5 also reports 272.
   Similarly, peripheral necrosis is reported as **98** in one section and **102** elsewhere.
   **A complete numerical cross-check of the manuscript, tables, figures, and supplementary material is essential.**
3. **NNH/causal interpretation remains problematic:**
   Although the response states that NNH has been reframed as an observational metric, parts of the revised manuscript still describe NNH as indicating complications attributable to **“therapeutic inertia”** and refer to **“preventable”** complications.
   These statements are inappropriate for an observational cohort and should be removed or completely rewritten.
4. **Statistical terminology requires further clarification:**
   The manuscript alternates between RR, OR, IRR, and aRR, and the definition of the outcome being modeled is not always clear. In particular, the reported aOR of 2.18 is sometimes interpreted as an independent “driver” of organ damage, which is stronger than an observational association.
   The authors should consistently distinguish **association from causation** and clearly identify the statistical model corresponding to each estimate.
5. **High-risk phenotype definition should be standardized:**
   The Methods appropriately define the high-risk phenotype as **both hypertension and obesity**, but the Abstract still uses slightly different threshold wording (BMI >30 and BP >140/90 rather than ≥30 and ≥140/90).
   This should be harmonized throughout.
6. **Study objectives and outcomes need better alignment:**
   The manuscript describes T2D prevalence/incidence, MACRE, renal decline, MASLD, peripheral necrosis, heart failure, and ischemic encephalopathy as outcomes. The hierarchy of primary versus secondary outcomes should be made more explicit to avoid selective emphasis on significant findings.

**Recommendation**

**Major Revision**

The manuscript has improved substantially and the authors have responded constructively to the previous reviewers' concerns. However, **the remaining numerical discrepancies—particularly the 52.3% versus 64.0% diabetes prevalence and the 272 versus 321 MACRE events—are sufficiently important that I would not recommend acceptance until they are fully reconciled.** The causal/therapeutic interpretation of NNH should also be corrected.

**Suggested final reviewer statement:**

*The revision has substantially improved the manuscript; however, important internal numerical inconsistencies remain, particularly regarding diabetes prevalence and the number of composite MACRE events. In addition, some causal interpretations of observational NNH estimates persist despite the authors' stated revisions. I recommend one further major revision focused primarily on complete numerical reconciliation, consistency of statistical terminology, and removal of residual causal language. Once these issues are satisfactorily addressed, the manuscript may be suitable for further consideration.*
