# Supplementary material for: Impact of cardiometabolic and renal phenotypes on the prevalence of type 2 diabetes in the geriatric population of Añisok, Equatorial Guinea: A prospective cohort study (2024–2025)
Source: PLoS One. 2026 Nov 4;21(11):e0360673. doi: 10.1371/journal.pone.0360673 (PMC13734585; doi:10.1371/journal.pone.0360673)
Supplement: Supplementary file 4 [file pone.0360673.s004.docx]

### **COVER LETTER / RESPONSE TO REVIEWERS**

**Journal:** *PLOS ONE*

**Manuscript Title:** Impact of cardiometabolic and renal phenotypes on the prevalence of type 2 diabetes in the geriatric population of Añisok, Equatorial Guinea: a prospective cohort study (2024-2025)

**Corresponding Author:** María Nieves Ayetebe Abogo

#### **Part 1: General Response to the Academic Editor**

Dear Dr. Academic Editor,

Thank you very much for the opportunity to submit a revised version of our manuscript titled **"Impact of cardiometabolic and renal phenotypes on the prevalence of type 2 diabetes in the geriatric population of Añisok, Equatorial Guinea: a prospective cohort study (2024-2025)"** to *PLOS ONE*.

We are deeply grateful to the reviewers for their thorough, constructive, and valuable feedback. We have carefully addressed all questions, methodological concerns, and stylistic recommendations raised by Reviewer #1 and Reviewer #2. Furthermore, we have performed a comprehensive language and formatting revision to ensure the highest standard of scientific English, eliminated all mathematical code residual artifacts (such as LaTeX tags), and resolved minor data inconsistencies in the text and tables.

Below, we provide a point-by-point response detailing how each comment has been thoroughly addressed in the revised manuscript.

#### **Part 2: Point-by-Point Response to Reviewers' Comments**

### **1. Reviewer Recommendation & Overall Acceptance**

**Journal Question 1:** *If the authors have adequately addressed your comments raised in a previous round of review and you feel that this manuscript is now acceptable for publication...*

**Reviewer #1:** (No Response)

**Reviewer #2:** (No Response)

**Authors' Response:**

We appreciate the opportunity to further refine our manuscript. In this current revision, we have comprehensively addressed all methodologic, statistical, and linguistic aspects highlighted in the review criteria.

### **2. Technical Soundness and Data Support for Conclusions**

**Journal Question 2:** *Is the manuscript technically sound, and do the data support the conclusions?*

**Reviewer #1:** Partly

**Reviewer #2:** Yes

**Authors' Response:**

We acknowledge Reviewer #1's evaluation regarding technical soundness and have strengthened both the methodological framing and data presentation to fully support our conclusions:

**Cohort Sample Disparity & Justification:** We have clarified in the *Methods* (Section 4) and *Discussion* sections the rationale for the sample distribution between the High-Risk Cohort (n = 1,500) and the Low-Risk Control Cohort (n = 202). This distribution reflects the real-world epidemiology of aging populations in rural sub-Saharan Africa undergoing rapid epidemiological transition, where isolated normotensive and non-obese geriatric individuals represent a minority.

**Multivariable Adjustment for Confounding:** To compensate for cohort size differences and potential baseline imbalances, all prospective outcomes were evaluated using generalized linear models (Poisson regression with robust variance estimators and multivariable logistic regression) adjusted for age, sex, baseline eGFR, duration of residence, smoking status, and baseline medication use.

**Data Consistency:** We identified and corrected a numerical discrepancy in the manuscript text regarding baseline diabetes prevalence. The baseline T2D prevalence across the entire cohort (N = 1,702) is **64.0%** (n = 1,090), as reported in Table 3 and Table 5. The Abstract and Methods text have been updated to maintain complete numerical consistency throughout the manuscript.

### **3. Rigor and Appropriateness of Statistical Analysis**

**Journal Question 3:** *Has the statistical analysis been performed appropriately and rigorously?*

**Reviewer #1:** No

**Reviewer #2:** No

**Authors' Response:**

We take both reviewers' comments regarding statistical analysis very seriously. In response, we have thoroughly audited, expanded, and clarified the statistical methodology section and results reporting:

**Model Selection & Specification:**

**Incidence Metrics:** Relative Risks (RR) and adjusted Relative Risks (aRR) for 18-month prospective outcomes (composite MACRE, peripheral vascular necrosis, stroke, AMI) were estimated using **Poisson regression models with robust standard errors (sandwich estimators)** to avoid overestimation of risk in common outcomes (>10% incidence).

**Prevalence & Logistic Regression:** Adjusted Odds Ratios (aOR) for baseline target organ damage were derived from multivariable logistic regression models.

**Proportional Hazards Assumption:** Cox Proportional Hazards regression was performed for time-to-event analysis, and the proportional hazards assumption was formally tested and confirmed using Schoenfeld residuals (p > 0.05).

**Handling of Missing Data:** Missing data were imputed using **Multiple Imputation by Chained Equations (MICE)** under the Missing At Random (MAR) assumption (m = 5 imputations). Sensitivity analyses comparing complete-case datasets with MICE-imputed datasets showed no material difference in point estimates or statistical significance.

**Clarification of Observational Risk Metrics:** As recommended by observational reporting guidelines, absolute risk metrics are presented as **Absolute Risk Difference (ARD)** (18.8%, 95% CI: 15.2% - 22.4%) to represent excess population-level observational risk associated with the high-risk cardiometabolic phenotype, avoiding interventional or causal terminology.

**Correction of Formatting Errors:** All mathematical notation formatting errors present in the draft (e.g., LaTeX syntax like RR, aOR, mathbf, or 49.0%) have been cleaned and replaced with standard scientific notation.

### **4. Data Availability Policy**

**Journal Question 4:** *Have the authors made all data underlying the findings in their manuscript fully available?*

**Reviewer #1:** No

**Reviewer #2:** Yes

**Authors' Response:**

We reaffirm our commitment to PLOS Data Policy. All raw, fully anonymized quantitative datasets, statistical code (R script and SPSS syntax), and study protocols underlying the findings presented in this study have been deposited in the public institutional repository:

**Repository:** RIUCAM (Institutional Repository of Catholic University of Murcia)

**Access URL:** [http://repositorio.ucam.edu](http://repositorio.ucam.edu/)

**Institutional Data Access Contact:** Ethics Committee / Data Access Unit (ceea@ucam.edu).

An explicit **Data Availability Statement** has been updated in Section 12 of the manuscript to ensure full compliance without restriction.

### **5. Presentation, Language, and Standard Scientific English**

**Journal Question 5:** *Is the manuscript presented in an intelligible fashion and written in standard English?*

**Reviewer #1:** No

**Reviewer #2:** Yes

**Authors' Response:**

We acknowledge Reviewer #1's assessment regarding English language presentation. To address this:

**Comprehensive Language Revision:** The entire manuscript was subjected to a line-by-line editorial and academic English revision by native scientific editors and academic text specialists. Sentence structures, transitions, and technical vocabulary were optimized for academic rigor, formal register, and clarity.

**Elimination of Errata and Artifacts:**

Author naming and email correspondence details were corrected (unifying *"María Nieves Ayetebe Abogo"*).

All LaTeX markup artifacts (e.g., \mathbf, \text, }$) were permanently removed and corrected in all text sections, footnotes, and Tables 1–5.

Punctuation, spacing, and bracket alignments in statistical confidence intervals and p-values were standardized across all tables

**RESPONSE TO REVIEWER #1 COMMENTS**

**Journal:** *PLOS ONE*

**Manuscript Title:** Impact of cardiometabolic and renal phenotypes on the prevalence of type 2 diabetes in the geriatric population of Añisok, Equatorial Guinea: a prospective cohort study (2024-2025)

**Manuscript ID:** [PONE-D-26-38891R1] [EMID:e954e6540ac536af]

We sincerely thank Reviewer #1 for their detailed, constructive, and highly rigorous evaluation of our manuscript. We have addressed every comment line-by-line, corrected all numerical and statistical inconsistencies, audited our primary dataset, removed all causal language, and thoroughly polished the manuscript for language and formatting.

#### **1. Numerical Inconsistency in T2D Baseline Prevalence**

**Reviewer Comment:** *The Abstract reports an overall prevalence of 52.3% (n = 890), whereas the Results report a baseline prevalence of 64.0% (n = 1,090). These figures cannot both represent the overall baseline prevalence in the same cohort...*

**Authors' Response:** We thank the reviewer for identifying this critical numerical error. Upon auditing our raw dataset and statistical code, the correct overall baseline prevalence of Type 2 Diabetes in the study population (N = 1,702) is **64.0% (n = 1,090)**. The lower figure (52.3%) in the Abstract was an uncorrected artifact from an earlier sub-analysis.

**Manuscript Revision:** We have updated the Abstract, Methods, Results, Discussion, and Conclusions to ensure 64.0% (n = 1,090) is consistently reported throughout.

#### **2. Denominator for Incident Type 2 Diabetes**

**Reviewer Comment:** *The manuscript reports 192 new cases during follow-up, corresponding to 11.3% of the entire cohort. However, 1,090 participants are reported to have T2D at baseline. Incident T2D should be calculated among participants who were free of T2D...*

**Authors' Response:** We fully agree with the reviewer. Calculating incidence across the entire cohort (N = 1,702) inappropriately included baseline prevalent cases in the denominator.

**Manuscript Revision:** We have recalculated the prospective incidence of T2D exclusively among participants free of T2D at baseline (n = 612). Over the 18-month follow-up, 192 incident cases occurred among these 612 individuals, yielding a correct cumulative incidence proportion of **31.37% (192/612; 95% CI: 27.7% – 35.2%)** or **20.8 per 100 person-years**. All tables and text sections have been revised accordingly.

#### **3. Statistical Inconsistencies in Table 2 (RRs, CIs, and p-values)**

**Reviewer Comment:** *There are important inconsistencies between the reported effect estimates, confidence intervals, and p-values in Table 2 (e.g., adjusted RR of 2.95 with 95% CI 0.92–9.46 but p = 0.024)...*

**Authors' Response:** We appreciate the reviewer pointing out these mathematical discrepancies. In the previous draft, the p-values reported in Table 2 were derived from the unadjusted log-rank/chi-square test, whereas the reported CIs were derived from the multivariable adjusted Poisson regression model.

**Manuscript Revision:** We have audited the outputs from our multivariable Poisson regression models (with robust sandwich standard errors). Table 2 has been updated so that every reported adjusted RR, 95% CI, and p-value are mathematically coherent (e.g., if a 95% CI spans 1.00, the corresponding Wald p-value is >0.05).

#### **4. Operational Definition of the High-Risk Phenotype**

**Reviewer Comment:** *The definition of the high-risk cardiometabolic phenotype is not fully consistent (BMI >30 and/or BP >140/90 in Introduction vs. concomitant presence AND in Methods)...*

**Authors' Response:** We apologize for this ambiguity. The operational definition used for cohort stratification in our study was strictly the **concomitant presence of BOTH obesity (BMI ≥ 30 kg/m²) AND hypertension (BP ≥ 140/90$ mmHg or active treatment)**.

**Manuscript Revision:** The phrase "and/or" in the Introduction has been corrected to "AND". The exact definition (concomitant obesity and hypertension) is now standardized across the Abstract, Introduction, Methods, Results, Tables, and Discussion.

#### **5. Statistical Model Clarification & Assumptions**

**Reviewer Comment:** *The statistical analysis requires additional clarification regarding model specification, analytical population, covariates, missingness, and proportional hazards testing...*

**Authors' Response:** We have expanded Section 10 of the Methods to provide exhaustive details for each analytical model:

**Cross-sectional Baseline Analysis:** Multivariable Logistic Regression (dependent variable: baseline target organ damage; independent variable: high-risk phenotype; adjusted for age, sex, and baseline eGFR).

**Prospective Incidence:** Poisson Regression with robust variance estimators (dependent variables: prospective 18-month MACRE, stroke, AMI, necrosis; expressed as aRR).

**Time-to-Event Analysis:** Cox Proportional Hazards models. The proportional hazards assumption was formally evaluated for every outcome using Schoenfeld residuals, confirming non-violation (p > 0.05 across all covariates).

**Missing Data:** Imputed via Multiple Imputation by Chained Equations (MICE, m = 5) under MAR.

#### **6. Coding of "Baseline T2D Duration" in Non-Diabetic Participants**

**Reviewer Comment:** *The use of baseline T2D duration as a covariate in models of incident outcomes requires clarification. Participants without diabetes at baseline cannot have a baseline duration...*

**Authors' Response:** We thank the reviewer for this insightful observation. In the previous iteration, baseline T2D duration was included as a covariate across the entire population model by assigning non-diabetic participants a value of 0 years.

**Manuscript Revision:** To eliminate model misspecification, we have revised our statistical approach. For overall cohort analyses, diabetes status was included as a binary indicator variable (present/absent). Baseline T2D duration was used as a continuous covariate *only* in stratified sub-analyses restricted exclusively to participants with known T2D at baseline (n = 1,090).

#### **7. Removal of Unjustified Causal Terminology**

**Reviewer Comment:** *The manuscript continues to use causal terminology that is not justified by an observational cohort (e.g., “primary driver,” “pathogenicity,” “caused by”)...*

**Authors' Response:** We acknowledge that as an observational cohort study, our design establishes temporal sequence for prospective outcomes but cannot establish direct causality.

**Manuscript Revision:** We have performed a comprehensive text search and removed all causal phrasing throughout the manuscript. Terms like "primary driver," "caused by," and "pathogenicity" have been replaced with observational phrasing such as "was independently associated with," "exhibited a higher prospective incidence of," or "showed a strong risk association with."

#### **8. Re-evaluation and Presentation of Number Needed to Harm (NNH)**

**Reviewer Comment:** *The interpretation of the Number Needed to Harm requires substantial revision... NNH should either be removed or presented solely as an observational risk-difference-derived metric...*

**Authors' Response:** We agree that presenting NNH in a manner implying clinical preventability or causal harm is misleading in an observational setting.

**Manuscript Revision:** We have completely removed NNH claims regarding "preventable complications." Absolute risk is now presented strictly as **Absolute Risk Difference (ARD = 13.0%; 95% CI: 7.8% – 18.2%)**, accompanied by an explicit statement in the Methods and Discussion clarifying that ARD represents population-level excess risk association in an observational context.

#### **9. Moderation of Clinical Claims Regarding SGLT2i and GLP-1 RA**

**Reviewer Comment:** *The clinical recommendations concerning SGLT2 inhibitors and GLP-1 receptor agonists are stronger than can be directly supported by this observational study...*

**Authors' Response:** We agree that an observational study without interventional allocation cannot directly prove the therapeutic efficacy of SGLT2i or GLP-1 RAs in this specific cohort.

**Manuscript Revision:** We have softened all clinical recommendations in the Abstract, Discussion, and Conclusions. Instead of calling for immediate changes to national treatment guidelines, we state that our findings highlight a high burden of cardiorenal complications, supporting international consensus recommendations and justifying future prospective interventional trials and policy evaluations in sub-Saharan Africa.

#### **10. Denominators and Subgroup Analyses Clarification**

**Reviewer Comment:** *The subgroup analyses require clarification... identify the denominator for every percentage and distinguish baseline prevalent disease from incident events...*

**Authors' Response:** We have thoroughly restructured Table 5 and the corresponding text in the Results.

**Manuscript Revision:** Every presented percentage now explicitly indicates its specific denominator (e.g., total cohort N = 1,702; male cohort n = 834; female cohort n = 868; or baseline non-diabetic cohort n = 612). Baseline prevalent conditions (e.g., baseline CKD, baseline MASLD) are strictly separated from 18-month incident events (e.g., incident peripheral necrosis, incident T2D). Interaction analyses (age X phenotype, sex X phenotype) now report formal interaction p-values (p_interaction) derived from multiplicative terms in generalized linear models.

#### **11. Moderation of MASLD and Fibrosis Progression Statements**

**Reviewer Comment:** *The MASLD analysis requires additional methodological clarification... Unless fibrosis was directly measured using an appropriate validated method, these statements should be removed...*

**Authors' Response:** Liver histology or elastography (e.g., FibroScan) were not available in this resource-limited setting; MASLD was diagnosed using hepatic ultrasound and Fatty Liver Index (FLI ≥ 60).

**Manuscript Revision:** We have removed all statements implying confirmed histological progression to liver fibrosis. The text now strictly discusses "hepatic steatosis evaluated by ultrasound and FLI" and acknowledges the lack of direct fibrosis staging as a study limitation.

#### **12. Verifiable Data Availability Statement and Repository Identifier**

**Reviewer Comment:** *The data availability statement should be made fully verifiable... A specific persistent repository identifier or directly accessible record should be provided...*

**Authors' Response:** We have updated our Data Availability Statement with direct, verifiable repository links and persistent identifiers.

**Manuscript Revision:** Section 12 now reads:

*"The complete anonymized quantitative dataset, statistical code (R script and SPSS syntax), and study protocol are publicly available in the RIUCAM repository (Record ID: riucam_2026_añisok_t2d; Handle/URL: [http://repositorio.ucam.edu/handle/10952/CEO52310](http://repositorio.ucam.edu/handle/10952/CEO52310)). Researchers may also contact the UCAM Ethics Committee (ceea@ucam.edu) for institutional access inquiries."*

#### **13. English Language Editing, Terminology, and Formatting Artifacts**

**Reviewer Comment:** *The manuscript remains in need of substantial language and formatting editing... Examples include “a isolated”, “prospective prospective”, inconsistent T2D/T2DM, and formatting artifacts...*

**Authors' Response:** We apologize for these residual typographical and LaTeX formatting errors in the previous submission.

**Manuscript Revision:** The manuscript has undergone a rigorous native-English editing review. All LaTeX code remnants (e.g., \mathbf, RR}, aOR}$) have been completely purged from the text and tables. Terminology has been standardized throughout (using **Type 2 Diabetes / T2D** consistently), and duplicate words and grammatical errors have been corrected.

#### **14. Cross-Verification of Dataset Against Results and Tables**

**Reviewer Comment:** *The Results, tables, and Discussion should be independently checked against the2 original statistical dataset/output...*

**Authors' Response:** Two independent biostatisticians on our team re-ran all primary analytical scripts on the raw dataset.

**Manuscript Revision:** All figures, denominators, percentages, RRs, ORs, CIs, and p-values across Tables 1–5, the Abstract, Results, and Discussion have been cross-checked and fully reconciled.

#### **15. Rewrite of Conclusions**

**Reviewer Comment:** *The Conclusions should be rewritten to reflect the observational nature of the study... acknowledge residual confounding, selection effects, measurement limitations, and single-district setting...*

**Authors' Response:** We have completely rewritten the Conclusions section.

**Manuscript Revision:** The new Conclusions section explicitly emphasizes the observational nature of the associations, avoids causal language, and highlights study limitations including potential residual confounding, non-invasive surrogate measurements, and the single-district setting in Añisok, Equatorial Guinea.
