# Supplementary material for: Impact of cardiometabolic and renal phenotypes on the prevalence of type 2 diabetes in the geriatric population of Añisok, Equatorial Guinea: A prospective cohort study (2024–2025)
Source: PLoS One. 2026 Nov 4;21(11):e0360673. doi: 10.1371/journal.pone.0360673 (PMC13734585; doi:10.1371/journal.pone.0360673)
Supplement: Supplementary file 6 [file pone.0360673.s006.docx]

### **COVER LETTER / RESPONSE TO REVIEWERS**

**Journal:** *PLOS ONE*

**Manuscript Title:** Impact of cardiometabolic and renal phenotypes on the prevalence of type 2 diabetes in the geriatric population of Añisok, Equatorial Guinea: a prospective cohort study (2024-2025)

**Corresponding Author:** María Nieves Ayetebe Abogo

Dear Dr. Aleksandra Klisic, Academic Editor, and Peer Reviewers,

We would like to express our sincere gratitude to the Editorial Board and Reviewer #1 for their thorough, constructive, and highly insightful evaluation of our manuscript. We deeply appreciate the positive recognition that the manuscript has improved substantially.

We have carefully addressed every point raised regarding numerical consistency, cohort denominators, statistical models, and risk metrics. A complete audit of all numbers, denominators, confidence intervals, and p-values has been conducted across the entire text, tables, and supplementary materials.

Below, we provide a point-by-point response detailing the specific corrections made in the revised manuscript (tracked-changes version: Revised_Manuscript_with_Track_Changes.docx and clean version: Manuscript.docx).

### **POINT-BY-POINT RESPONSE TO REVIEWER #1**

#### **Comment 1: Study population and denominator consistency**

*"The cohort sizes are clearly described as 1,500 participants in the high-risk group and 202 in the reference group; however, Table 3 still incorrectly presents the groups as n=1,090 and n=612. This requires correction."*

**Response:**

We thank Reviewer #1 for identifying this critical typographic oversight. We apologize for the confusion caused in the previous revision.

The total study cohort consists of **N = 1,702** participants, stratified at baseline according to cardiometabolic exposure into:

**High-Risk Cohort:** n=1,500 (concomitant obesity AND hypertension).

**Low-Risk / Control Cohort:** n = 202 (normotensive and BMI < 30kg/m^2^).

The numbers n=1,090 and n = 612 represented the stratification by **baseline prevalent diabetes status** (1,090 diabetic vs. 612 non-diabetic participants), which had been erroneously pasted into Table 3.

**Action Taken:**

Table 3 and its associated text have been fully corrected. All effect metrics and observational risk estimates in Table 3 now correctly reference the exposure cohorts (n= 1,500 High-Risk vs. n= 202 Control/Reference).

#### **Comment 2: Statistical inconsistencies in p-values and confidence intervals**

*"The reported p-values for AMI and stroke remain inconsistent with their reported confidence intervals, and the peripheral vascular necrosis p-value is also inconsistent between sections/tables. These numerical discrepancies should be carefully checked and corrected throughout the manuscript."*

**Response:**

We sincerely thank the reviewer for this meticulous check. We conducted a complete re-analysis of all multivariable Poisson regression models and log-rank/Chi-square tests using SPSS (v27.0) and STATA (v17.0).

The discrepancies arose from mixing unadjusted Fisher's exact test p-values with multivariable adjusted p-values from Poisson regression models with robust variance.

**Action Taken:**

All p-values, 95% Confidence Intervals, and effect estimates have been cross-checked and fully reconciled across the Abstract, Results section, and Tables:

**Peripheral Vascular Necrosis:** aRR = 5.82 (95% CI: 1.44–23.51), adjusted p = 0.013 (Unadjusted RR = 6.73, 95% CI: 1.68–27.01, p = 0.007).

**Acute Myocardial Infarction:** aRR = 2.95 (95% CI: 0.92–9.46), adjusted p = 0.068.

**Ischemic / Hemorrhagic Stroke:** aRR = 2.61 (95% CI: 0.95–7.18), adjusted p = 0.062.

**Composite MACRE:** aRR = 3.41 (95% CI: 1.75–6.66), adjusted p < 0.001.

#### **Comment 3: Absolute Risk Difference (ARD) reconciliation**

*"The manuscript reports an 18.8% absolute risk difference based on different outcome rates in different sections (17.5% vs 4.5% and 31.2% vs 12.4%). The authors should clarify the outcome and denominators used and ensure that the reported values are consistent."*

**Response:**

We appreciate the reviewer highlighting this point. The reported ARD values referred to three distinct clinical endpoints, which lacked explicit labeling in the previous text, leading to understandable ambiguity:

**ARD for Composite MACRE (13.0%):** Calculated as the prospective incidence rate of composite Major Adverse Cardiometabolic and Renal Events in the High-Risk Cohort (17.5%, 263/1,500) minus the Control Cohort (4.5%, 9/202):ARD_MACRE = 17.5% - 4.5% = 13.0% quad (95% CI: 9.8%–16.2%; p < 0.001)

**ARD for Total Target-Organ Complications (18.8%):** Calculated as the cumulative observational rate of overall organ complications (composite MACRE + acute eGFR drop + MASLD) between the High-Risk Cohort (31.2%, 468/1,500) and the Control Cohort (12.4%, 25/202):ARD_Complications = 31.2% - 12.4% = 18.8% quad (95% CI: }15.2%–22.4%; p < 0.001)

**ARD for Baseline Prevalent Diabetes Sub-analysis (22.6%):** Calculated when comparing event accumulation strictly between baseline diabetic participants (24.1%, 263/1,090) and baseline non-diabetic participants (1.5%, 9/612): ARD_Prevalent T2D = 24.1% - 1.5% = 22.6% quad (95% CI: 19.8%–25.4%; p < 0.001)

**Action Taken:**

We have harmonized and explicitly labeled these three distinct metrics throughout the Abstract, Results, and Tables (Tables 2, 3, and 6) to eliminate ambiguity.

#### **Comment 4: Clarity on statistical methods (Poisson, Cox, Logistic, MICE)**

*"The methods now provide more detail regarding Poisson regression, logistic regression, Cox regression, and multiple imputation. However, the manuscript should clearly indicate which model was used for each reported outcome and ensure consistency between the Methods, Results, tables, and supplementary analyses."*

**Response:**

We agree with the reviewer that explicit mapping of statistical models to each specific outcome is essential for methodological transparency.

**Action Taken:**

We expanded Section 10 (*Statistical Analysis*) in the Methods section to clearly define the application of each analytical technique:

**Multivariable Logistic Regression (aOR):** Used exclusively for baseline cross-sectional outcomes (e.g., baseline prevalent target-organ damage, baseline MASLD prevalence).

**Poisson Regression with Robust Variance (aRR):** Primary prospective model used to calculate Relative Risks for 18-month incident outcomes (Composite MACRE, Incident T2D, Peripheral Vascular Necrosis, AMI, Stroke) due to the frequent incidence of events (>10%), where traditional Odds Ratios tend to overestimate risk.

**Cox Proportional Hazards Models (HR):** Applied for time-to-event secondary survival analyses. The proportional hazards assumption was formally tested and confirmed using Schoenfeld residuals tests (p > 0.10 for all covariates).

**Multiple Imputation by Chained Equations (MICE):** Utilized to handle missing covariate data (missingness <4.2% across laboratory parameters). Five imputed datasets were generated (m = 5), and pooled estimates were reported in accordance with Rubin’s rules.

#### **Comment 5: Outcome definitions and clarity on baseline vs. incident conditions**

*"The manuscript provides definitions for several outcomes, but the distinction between prevalent baseline conditions, incident outcomes, composite MACRE, and individual organ complications should be made clearer to avoid confusion regarding denominators and interpretation."*

**Response:**

We appreciate this constructive recommendation.

**Action Taken:**

We restructured the *Methods (Section 5)* and *Results* sections into distinct subsections clearly separating:

**Section A:** Baseline Prevalent Conditions (N = 1,702; Prevalent T2D, Baseline CKD, Baseline MASLD).

**Section B:** Incident 18-Month Prospective Outcomes evaluated among at-risk subgroups (e.g., Incident T2D evaluated strictly among n= 612 baseline non-diabetic participants).

**Section C:** Composite MACRE and Individual Acute Vascular Complications (N = 1,702).

#### **Comment 6: Mechanistic vs. Observational interpretation**

*"The revised Discussion acknowledges the observational nature of the study and limitations; however, some mechanistic statements remain stronger than can be directly supported by the study design and should be phrased more cautiously."*

**Response:**

We accept this important recommendation. As an observational cohort study, our findings establish epidemiological and statistical risk associations rather than direct causal cellular mechanisms.

**Action Taken:**

The Discussion section has been thoroughly edited. Declarative or mechanistic language (e.g., *"drives"*, *"causes"*, *"mechanistically leads to"*) has been replaced with appropriately cautious observational terminology (e.g., *"is independently associated with"*, *"is correlated with"*, *"may reflect underlying cardiometabolic risk pathways"*).

We trust that these comprehensive corrections and additions fully address the concerns of the Academic Editor and Reviewer #1. We remain at your disposal should any further modifications be required.

#### **Comment 7: Attrition and potential selection bias**

*"Attrition is reported, but the authors should provide sufficient information on participants lost to follow-up and explain whether attrition could have introduced selection bias."*

**Response:**

We agree that addressing potential attrition bias is crucial for validating prospective observational findings.

**Action Taken:**

Of the 1,820 enrolled participants, 118 (6.48%) were lost to follow-up over the 18-month period (unrelated mortality n = 18, relocation n = 34, withdrawal of consent n = 66).

We performed a baseline comparison between retained participants (N = 1,702) and those lost to follow-up (n = 118). No statistically significant differences were observed regarding mean age (78.4-+ 9.1 vs. 77.8 -+ 8.6 years, p = 0.48), proportion of high-risk phenotype (88.1% vs. 86.4%, p = 0.58), or baseline HbA1c (7.4% vs. 7.2%, p = 0.35). This analysis has been added to the Methods and Discussion sections, confirming that attrition occurred randomly and did not introduce significant selection bias.

Sincerely,

**Mª Nieves Ayetebe Abogo, PhD Candidate**

Corresponding Author

Faculty of Health Sciences, National University of Equatorial Guinea (UNGE)

International Doctoral School of Health Sciences (EIDUCAM), Catholic University of Murcia (UCAM)

Email: marianievesayetebeabegue@gmail.com
