# Supplementary material for: Impact of cardiometabolic and renal phenotypes on the prevalence of type 2 diabetes in the geriatric population of Añisok, Equatorial Guinea: A prospective cohort study (2024–2025)
Source: PLoS One. 2026 Nov 4;21(11):e0360673. doi: 10.1371/journal.pone.0360673 (PMC13734585; doi:10.1371/journal.pone.0360673)
Supplement: Supplementary file 7 [file pone.0360673.s007.docx]

I re-reviewed the revised manuscript, focusing on whether the previous reviewer concerns were actually resolved and whether any important inconsistencies remain.

**Manuscript Title:**
**Impact of cardiometabolic and renal phenotypes on the prevalence of type 2 diabetes in the geriatric population of Añisok, Equatorial Guinea: a prospective cohort study (2024–2025)**

**Reviewer Comments**

The manuscript has improved substantially and most of the previous concerns regarding cohort denominators, statistical methods, outcome definitions, and risk estimates have been addressed. However, a few important issues remain:

1. **Major denominator inconsistency remains.** In the Results/Table 6 section, the high-risk and reference groups are still reported as **n=1,090 and n=612**, with event rates of 24.1% versus 1.5%. These are actually the baseline diabetic/non-diabetic groups, not the cardiometabolic exposure cohorts (n=1,500 and n=202). This conflicts with Table 2 and the Methods and should be corrected throughout.
2. **Interpretation of attrition should be more cautious.** The absence of statistically significant baseline differences between participants retained and lost to follow-up does not establish that attrition “occurred randomly.” The authors should state that no significant baseline differences were detected and acknowledge that residual attrition bias cannot be excluded.
3. **Table 6 and its accompanying text require careful reconciliation.** The denominators, phenotype labels, percentages, and event estimates should be checked against the primary exposure definition and Table 2 to ensure that baseline diabetes status is not being confused with cardiometabolic phenotype.
4. **Terminology should be standardized.** The manuscript alternates between “MACRE” described as cardiometabolic and renal events and elsewhere as cardiovascular and renal events. Please use one consistent definition and terminology throughout.
5. **Minor editorial revision:** Correct remaining typographical/formatting problems, particularly inconsistent spacing around statistical values, symbols, and confidence intervals, before publication.

**Recommendation: Minor Revision.**
