# Supplementary material for: Impact of cardiometabolic and renal phenotypes on the prevalence of type 2 diabetes in the geriatric population of Añisok, Equatorial Guinea: A prospective cohort study (2024–2025)
Source: PLoS One. 2026 Nov 4;21(11):e0360673. doi: 10.1371/journal.pone.0360673 (PMC13734585; doi:10.1371/journal.pone.0360673)
Supplement: Supplementary file 8 [file pone.0360673.s008.docx]

### **COVER LETTER / RESPONSE TO REVIEWERS**

**Journal:** *PLOS ONE*

**Re:** Submission of Revised Manuscript

Manuscript ID: PONE-D-26-38891R3 - EMID:ec61207bc7435354

**Manuscript Title:** Impact of cardiometabolic and renal phenotypes on the prevalence of type 2 diabetes in the geriatric population of Añisok, Equatorial Guinea: a prospective cohort study (2024-2025)

**Corresponding Author:** María Nieves Ayetebe Abogo

Dear Dr. Aleksandra Klisic, Academic Editor, and Peer Reviewers,

Thank you for granting us the opportunity to submit a revised version of our manuscript to *PLOS ONE*. We sincerely appreciate the constructive feedback and thorough evaluations provided by Reviewer 1 and Reviewer 2. Their insights have been invaluable in enhancing the methodological rigor, statistical clarity, and overall precision of our work.

Below, we provide a point-by-point response detailing how each comment and concern has been rigorously addressed in the revised manuscript. All changes made in the clean and tracked versions of the manuscript are identical.

### **PART 1: RESPONSE TO REVIEWER 1 (ROUND 1 COMMENTS)**

**Comment 1.1:** *Provide a CONSORT/STROBE style flow diagram at participant level and a pre-specified data dictionary... Replace Table 6 with a properly labeled diabetes status subgroup table or remove it from the primary inferential narrative. Recalculate each estimate...*

**Response 1.1:** Thank you for this critical recommendation. We have incorporated a comprehensive STROBE-compliant participant flow diagram detailing screening (N=2,145), eligibility (n=2,012), enrollment (n=1,820), loss to follow-up (n=118), and final analytical cohort (N=1,702). Furthermore, we have revised Table 6 to serve strictly as a secondary subgroup analysis stratified by baseline prevalent diabetes status (n=1,090 diabetic vs. n=612 non-diabetic), separating it clearly from the primary exposure cohort narrative (n=1,500 high-risk vs. n=202 control). All estimates across the abstract, main text, tables, and supplementary files have been fully reconciled.

**Comment 1.2:** *Define each estimator precisely: first incident MACRE component, recurrent events if applicable, baseline target organ damage, incident eGFR decline, incident MASLD, and vascular necrosis. Indicate index date, censoring date, and handling of competing risks...*

**Response 1.2:** We have expanded Section 5 & 6 of the Methods to explicitly define all clinical estimators and operational timelines. The index date was set as the enrollment/baseline evaluation date (January–June 2024). Censoring dates were set at the time of last completed quarterly evaluation or study end (December 2025). Non-endpoint mortality (n=18) was explicitly modeled as a competing risk rather than simple loss to follow-up. Composite endpoints have been broken down, and component-specific results are now explicitly presented.

**Comment 1.3:** *Provide a comprehensive model table mapping outcome to model, estimator, link, variance estimator, covariates, missing data approach, follow-up scale, and reference category. Use appropriate primary effect measure...*

**Response 1.3:** As requested, we have added a comprehensive Model Summary Table in the Supplementary Material (S1 Table) mapping every endpoint to its corresponding regression framework (multivariable Poisson regression with robust error variance for common prospective outcomes, yielding adjusted Relative Risks [aRR]; logistic regression for cross-sectional baseline parameters, yielding adjusted Odds Ratios [aOR]). Missing baseline laboratory data (<4.2%) were handled using Multiple Imputation by Chained Equations (MICE, $m=5$). Exact estimates, 95% CIs, and p-values are reported consistently throughout the text and tables.

**Comment 1.4:** *Provide a supplementary audit table containing numerators, denominators, percentages, unadjusted estimates, adjusted estimates, 95% CIs, p-values, and exact analytical sample... Include reproducible code.*

**Response 1.4:** A complete Supplementary Audit Table (S2 Table) and an unredacted data dictionary have been prepared and uploaded to the institutional repository alongside the reproducible analytical code (R script and SPSS syntax).

**Comment 1.5:** *Predefine a minimally sufficient adjustment set using domain knowledge... Distinguish confounders from mediators... provide sensitivity analyses.*

**Response 1.5:** We have updated the Methods section to clarify that covariate selection was guided by clinical domain knowledge (age, sex, residency duration, baseline eGFR, smoking, and baseline medication). Potential mediators (such as post-baseline glycemic trajectory) were excluded from baseline adjustment sets to avoid over-adjustment bias. Absolute risk differences (ARD) with 95% CIs have also been explicitly reported alongside relative effect estimates.

**Comment 1.6:** *Show a complete attrition table with standardized mean differences (SMD) and missing data for all key baseline variables...*

**Response 1.6:** An attrition table comparing retained participants (N=1,702) and those lost to follow-up (n=118) including SMDs has been added to the Results section. We have also revised our interpretation of attrition (see Response to Reviewer 2, Comment 2).

### **PART 2: RESPONSE TO REVIEWER 2 (RE-REVIEW COMMENTS)**

**Comment 2.1 (Major denominator inconsistency):** *In the Results/Table 6 section, the high-risk and reference groups are still reported as n=1,090 and n=612, with event rates of 24.1% versus 1.5%. These are actually the baseline diabetic/non-diabetic groups, not the cardiometabolic exposure cohorts (n=1,500 and n=202). This conflicts with Table 2 and the Methods and should be corrected throughout.*

**Response 2.1:** We sincerely thank Reviewer 2 for pointing out this critical distinction. We apologize for the confusion caused by overlapping denominator labels. We have thoroughly corrected and re-labeled the manuscript to ensure complete consistency:

**1.-Primary Exposure Cohorts:** High-Risk Cohort (n=1,500, concomitant obesity AND hypertension) vs. Low-Risk / Control Cohort (n=202, non-obese, normotensive). Table 2 and primary text narrative reflect these denominators strictly.

**2.-Secondary Subgroup Analysis (Table 6):** Table 6 and its accompanying text have been explicitly re-titled and restructured as a secondary subgroup analysis based on *Baseline Prevalent Diabetes Status* (n=1,090 with T2D vs. n=612 non-diabetic at baseline). We have ensured that baseline diabetes status is nowhere confused with the primary cardiometabolic risk exposure cohorts.

**Comment 2.2 (Interpretation of attrition):** *The absence of statistically significant baseline differences between participants retained and lost to follow-up does not establish that attrition “occurred randomly.” The authors should state that no significant baseline differences were detected and acknowledge that residual attrition bias cannot be excluded.*

**Response 2.2:** We completely agree with this nuanced interpretation. We have revised the Results (Section 1) and Discussion sections to remove the statement that attrition "occurred randomly." The revised text now reads:

*"Baseline comparison between retained participants (N=1,702) and those lost to follow-up (n=118) detected no statistically significant differences regarding mean age, proportion of high-risk phenotype, or baseline HbA1c. Nevertheless, while major baseline imbalances were not detected, residual attrition bias cannot be entirely excluded."*

**Comment 2.3 (Table 6 reconciliation):** *Table 6 and its accompanying text require careful reconciliation. The denominators, phenotype labels, percentages, and event estimates should be checked against the primary exposure definition and Table 2 to ensure that baseline diabetes status is not being confused with cardiometabolic phenotype.*

**Response 2.3:** Table 6 and all corresponding text in the Results and Discussion have been fully reconciled and cross-checked against Table 2 and the Methods section. Table 6 now explicitly states in its title and column headers that it represents a *Secondary Subgroup Analysis Stratified by Baseline Prevalent Diabetes Status* (n=1,090 vs. n=612).

**Comment 2.4 (Terminology standardization):** *The manuscript alternates between “MACRE” described as cardiometabolic and renal events and elsewhere as cardiovascular and renal events. Please use one consistent definition and terminology throughout.*

**Response 2.4:** Thank you for highlighting this inconsistency. We have standardized the definition throughout the entire manuscript, title, tables, and abstract to: **"Major Adverse Cardiometabolic and Renal Events (MACRE)"**.

**Comment 2.5 (Minor editorial revision):** *Correct remaining typographical/formatting problems, particularly inconsistent spacing around statistical values, symbols, and confidence intervals, before publication.*

**Response 2.5:** A comprehensive line-by-line editorial proofreading has been conducted. We have standardized spacing around statistical values, symbols, and confidence intervals throughout the manuscript (e.g., ensuring consistent formatting such as p < 0.001, 95% CI: 1.75-6.66, and proper spacing before units).

Sincerely,

**Mª Nieves Ayetebe Abogo, PhD Candidate**

Corresponding Author

Faculty of Health Sciences, National University of Equatorial Guinea (UNGE)

International Doctoral School of Health Sciences (EIDUCAM), Catholic University of Murcia (UCAM)

Email: marianievesayetebeabegue@gmail.com
