# Supplementary material for: Impact of cardiometabolic and renal phenotypes on the prevalence of type 2 diabetes in the geriatric population of Añisok, Equatorial Guinea: A prospective cohort study (2024–2025)
Source: PLoS One. 2026 Nov 4;21(11):e0360673. doi: 10.1371/journal.pone.0360673 (PMC13734585; doi:10.1371/journal.pone.0360673)
Supplement: Peer review history

# Peer review: Decision Letter 0

**DOI:** 10.1371/journal.pone.0360673.r001

**Authors:** Aleksandra Klisic

25 Aug 2026

Dear Dr. Ayetebe Abogo,

Thank you for submitting your manuscript to PLOS One. After careful consideration, we feel that it has merit but does not fully meet PLOS One’s publication criteria as it currently stands. Therefore, we invite you to submit a revised version of the manuscript that addresses the points raised during the review process.

Please submit your revised manuscript by Oct 24 2026 11:59PM. If you will need more time than this to complete your revisions, please reply to this message or contact the journal office at plosone@plos.org. When you're ready to submit your revision, log on to https://www.editorialmanager.com/pone/ and select the 'Submissions Needing Revision' folder to locate your manuscript file.

- A letter that responds to each point raised by the academic editor and reviewer(s). You should upload this letter as a separate file labeled 'Response to Reviewers'.
- A marked-up copy of your manuscript that highlights changes made to the original version. You should upload this as a separate file labeled 'Revised Manuscript with Track Changes'.
- An unmarked version of your revised paper without tracked changes. You should upload this as a separate file labeled 'Manuscript'.

If you would like to make changes to your financial disclosure, please include your updated statement in your cover letter. Guidelines for resubmitting your figure files are available below the reviewer comments at the end of this letter.

If applicable, we recommend that you deposit your laboratory protocols in protocols.io to enhance the reproducibility of your results. Protocols.io assigns your protocol its own identifier (DOI) so that it can be cited independently in the future. For instructions see: https://journals.plos.org/plosone/s/submission-guidelines#loc-laboratory-protocols. Additionally, PLOS One offers an option for publishing peer-reviewed Lab Protocol articles, which describe protocols hosted on protocols.io. Read more information on sharing protocols at https://plos.org/protocols?utm_medium=editorial-email&utm_source=authorletters&utm_campaign=protocols.

As the corresponding author, your ORCID iD is verified in the submission system and will appear in the published article. PLOS supports the use of ORCID, and we encourage all coauthors to register for an ORCID iD and use it as well. Please encourage your coauthors to verify their ORCID iD within the submission system before final acceptance, as unverified ORCID iDs will not appear in the published article. *Only*  the individual author can complete the verification step; PLOS staff *cannot*  verify ORCID iDs on behalf of authors.

We look forward to receiving your revised manuscript.

Kind regards,

Aleksandra Klisic

Academic Editor

PLOS One

Journal Requirements:

When submitting your revision, we need you to address these additional requirements.

1. Please ensure that your manuscript meets PLOS ONE's style requirements, including those for file naming. The PLOS ONE style templates can be found at

https://journals.plos.org/plosone/s/file?id=wjVg/PLOSOne_formatting_sample_main_body.pdf and

https://journals.plos.org/plosone/s/file?id=ba62/PLOSOne_formatting_sample_title_authors_affiliations.pdf

2. In the online submission form you indicate that your data is not available for proprietary reasons and have provided a contact point for accessing this data. Please note that your current contact point is a co-author on this manuscript. According to our Data Policy, the contact point must not be an author on the manuscript and must be an institutional contact, ideally not an individual. Please revise your data statement to a non-author institutional point of contact, such as a data access or ethics committee, and send this to us via return email. Please also include contact information for the third party organization, and please include the full citation of where the data can be found.

3. Your ethics statement should only appear in the Methods section of your manuscript. If your ethics statement is written in any section besides the Methods, please delete it from any other section.

4. If the reviewer comments include a recommendation to cite specific previously published works, please review and evaluate these publications to determine whether they are relevant and should be cited. There is no requirement to cite these works unless the editor has indicated otherwise.

[Note: HTML markup is below. Please do not edit.]

Reviewers' comments:

Reviewer's Responses to Questions

**Comments to the Author**

1. Is the manuscript technically sound, and do the data support the conclusions?

Reviewer #1: Partly

Reviewer #2: No

**********

2. Has the statistical analysis been performed appropriately and rigorously?

Reviewer #1: No

Reviewer #2: No

**********

3. Have the authors made all data underlying the findings in their manuscript fully available?

The PLOS Data policy

Reviewer #1: No

Reviewer #2: No

**********

4. Is the manuscript presented in an intelligible fashion and written in standard English?

Reviewer #1: No

Reviewer #2: No

**********

Reviewer #1: This manuscript addresses an important and under-studied topic in an older population in Equatorial Guinea. The relatively large sample, prospective follow-up, and community-based recruitment are strengths. However, several methodological and statistical issues require clarification before the manuscript can be considered for publication.

Major Comments:-

Study population: The Methods state that confirmed T2DM was an inclusion criterion, but the Results report T2DM prevalence and include participants with HbA1c values consistent with no diabetes. Please clarify the study population and revise the eligibility criteria accordingly.

Definition of high-risk phenotype: The Methods use BMI >30 kg/m² and BP >140/90 mmHg, while the Results use “and/or.” Please provide one clear and consistent definition throughout the manuscript.

Numerical inconsistencies: There are important discrepancies between the tables and text. For example, Table 2 reports 100 peripheral necrosis events, whereas Table 5 reports 304 cases when the male and female numbers are combined. Please carefully check and correct all tables and results.

Risk estimates: The reported ARI of 18.8% does not appear to correspond to the MACRE rates reported in Table 2. Please recalculate and clarify the ARI and NNH. The interpretation of NNH should also avoid implying that the complications were preventable or caused by therapeutic inertia.

Statistical analysis: Please provide clearer information on the statistical models used to calculate RR, OR, and IRR, including the variables included in each model and the reference groups.

Time-to-event analysis: The Methods mention Kaplan–Meier and Cox regression, but corresponding results are not presented. Please provide the relevant hazard ratios and confidence intervals, or revise the Methods if these analyses were not performed.

Renal outcomes: Please clearly define how renal impairment/renal failure was diagnosed, including the eGFR calculation, measurements used, and timing of assessment.

MASLD assessment: Please provide more details on the diagnostic criteria, ultrasound assessment, and use of the Fatty Liver Index.

Missing data: Please provide additional information on the MICE procedure, including the number of imputations and variables included in the imputation model.

Causal interpretation: As this is an observational study, causal statements should be moderated. Terms such as “drives,” “preventable,” and “caused by therapeutic inertia” should be replaced with language describing associations.

Treatment recommendations: The study did not evaluate GLP-1 receptor agonists or SGLT2 inhibitors. Therefore, recommendations regarding their implementation should be presented cautiously and clearly separated from the findings of this study.

Primary outcome: Please clearly identify the primary outcome and distinguish it from secondary and exploratory analyses.

Participant flow: The numbers reported in the Methods and Results are not fully consistent. Please reconcile the population numbers and provide a clear participant flow diagram.

Baseline characteristics: Please provide additional important baseline variables, particularly diabetes status, diabetes duration, medications, smoking, renal function, and cardiovascular comorbidities.

Tables: Please carefully review Tables 2 and 5, particularly the peripheral necrosis data, and ensure that all denominators and percentages are correct.

The study has potential value, but the current manuscript requires substantial revision. The main priorities are to clarify the study population and exposure definitions, correct the numerical inconsistencies, improve the statistical reporting, and moderate

Reviewer #2: This manuscript addresses an important public-health issue by examining the relationship between cardiometabolic risk phenotypes and type 2 diabetes and related complications among older adults in Añisok, Equatorial Guinea. The topic is relevant and the cohort is potentially valuable. However, several methodological and interpretive issues should be addressed before the manuscript can be considered for publication.

The most important concern is the consistency between the study design, eligibility criteria, and primary outcome. The manuscript describes a prospective cohort, but the outcome is presented as T2DM prevalence, while the eligibility criteria appear to require confirmed T2DM. The authors should clearly state whether participants were enrolled irrespective of diabetes status and distinguish prevalent diabetes at baseline from incident diabetes during follow-up.

The definition of the high-risk cardiometabolic phenotype also appears inconsistent, being described in different sections using “and” versus “and/or” for obesity and hypertension. This should be standardized throughout the manuscript. In addition, the authors should clearly distinguish conditions present at baseline from complications that developed during follow-up.

The interpretation of therapeutic inertia and the reported number needed to harm (NNH) should be substantially revised. Because this is an observational cohort, the findings demonstrate associations and cannot establish that therapeutic inertia caused the observed complications. The NNH should therefore not be interpreted as the number of patients who would develop preventable complications because of inadequate treatment.

The definition of the renal outcome also requires clarification. A >30% decline in GFR should not automatically be described as acute renal failure. The authors should specify the method used to estimate eGFR, baseline renal status, timing of measurements, and criteria used to distinguish acute kidney injury from chronic renal deterioration.

The statistical analysis should be clarified. The covariates included in adjusted models appear inconsistent between the Methods and Results. The authors should provide a clearly prespecified adjustment strategy and explain the rationale for selecting confounders. The sample-size calculation should also be linked explicitly to the stated primary endpoint.

Finally, several conclusions appear stronger than warranted by the observational design. Terms such as “driver,” “caused,” or statements attributing complications to therapeutic inertia should be replaced by appropriately cautious language such as “associated with” or “associated with increased risk.”

Additional comments

The authors should reconcile all participant numbers across the Methods, Results, tables, and participant-flow diagram and provide clear definitions for MACRE and each of its components. Major effect estimates should preferably be accompanied by 95% confidence intervals. The manuscript would also benefit from a careful editorial review for terminology and consistency.

I did not identify an obvious concern regarding dual publication or publication ethics from the material reviewed. Nevertheless, the authors should ensure that the study has appropriate ethical approval, informed consent procedures, and transparent reporting of participant recruitment and follow-up.

Overall recommendation: Major Revision. The study has potential value, but clarification of the cohort design, outcome definitions, statistical analysis, and causal interpretation is necessary before the validity of the principal findings can be adequately assessed.

**********

what does this mean?). If published, this will include your full peer review and any attached files.

If you choose “no”, your identity will remain anonymous but your review may still be made public.

**Do you want your identity to be public for this peer review?**  For information about this choice, including consent withdrawal, please see our Privacy Policy

Reviewer #1: **Yes:**  Malak M Alagoury

Reviewer #2: No

**********

[NOTE: If reviewer comments were submitted as an attachment file, they will be attached to this email and accessible via the submission site. Please log into your account, locate the manuscript record, and check for the action link "View Attachments". If this link does not appear, there are no attachment files.]

To ensure your figures meet our technical requirements, please review our figure guidelines: https://journals.plos.org/plosone/s/figures

You may also use PLOS’s free figure tool, NAAS, to help you prepare publication quality figures: https://journals.plos.org/plosone/s/figures#loc-tools-for-figure-preparation.

NAAS will assess whether your figures meet our technical requirements by comparing each figure against our figure specifications.

Attachment Submitted filename: Reviewer Report cardiembolic.docx

# Author comment: Author response to Decision Letter 1

**DOI:** 10.1371/journal.pone.0360673.r002

8 Sep 2026

RESPONSE TO REVIEWERS AND EDITORIAL BOARD

Manuscript Title: Prospective Evaluation of Type 2 Diabetes Prevalence, Incident Cardiometabolic and Renal Events, and Target Organ Damage in a Geriatric Cohort from Añisok, Equatorial Guinea

Manuscript ID: PONE-D-26-38891

Journal: PLOS ONE

OVERVIEW AND GENERAL RESPONSE

We extend our sincere gratitude to the Academic Editor and the Reviewers for their meticulous, constructive, and highly valuable evaluation of our manuscript. We fully acknowledge the concerns raised regarding technical soundness, statistical rigor, data availability, and language clarity.

In response to the "Major Revision" decision, we have performed a comprehensive revision of the manuscript. Specifically, we have:

Standardized and unified all participant counts across the text, tables, and methodological descriptions (N = 1,702 total; n = 1,500 high-risk cohort; n = 202 low-risk control cohort).

Provided explicit operational definitions for major adverse cardiometabolic and renal events (MACRE) and each of its individual components.

Reported 95% Confidence Intervals (95% CIs) for all primary effect estimates in the revised Tables (Tables 2 and 5).

Clarified the observational framework for risk metrics (Number Needed to Harm [NNH] and Absolute Risk Increase [ARI]), explicitly removing causal claims.

Incorporated required PLOS ONE compliance statements, including Data Availability, Researcher Reflexivity, Sex as a Biological Factor, and PPI.

Extensively revised the English terminology, syntax, and scientific style to ensure standard PLOS ONE academic quality.

RESPONSE TO STRUCTURED REVIEW EVALUATION CRITERIA

1. Technical Soundness and Support of Conclusions

Reviewer #1 Rating: Partly | Reviewer #2 Rating: No

Authors' Response:

We have thoroughly revised the study design, population eligibility, and outcome reporting to ensure absolute technical soundness:

Sample Size and Cohort Balance: We have clarified that the total analytical cohort consists of N = 1,702 community-dwelling older adults (aged 60–100 years). The exposure cohort comprises n = 1,500 high-risk participants (concomitant hypertension and obesity), while the reference control cohort comprises n = 202 low-risk participants (normotensive and non-obese). We have addressed this imbalance in the Limitations section, noting that it reflects the high background prevalence of cardiometabolic risk in this aging population, and confirmed that multivariable models adjusted for baseline imbalances.

Exclusion Criteria Refinement: To eliminate acute confounding, we explicitly added exclusion criteria for active acute infectious diseases at sampling time (e.g., clinical malaria, acute sepsis), terminal illness (<6 months life expectancy), Type 1 diabetes, and withdrawal of consent.

MACRE Definition: MACRE was defined as a composite outcome comprising: (1) cardiovascular mortality, (2) non-fatal myocardial infarction, (3) stroke, and (4) severe peripheral vascular events/necrosis.

2. Statistical Analysis Rigor

Reviewer #1 Rating: No | Reviewer #2 Rating: No

Authors' Response:

We have completely overhauled the statistical presentation and methodology to meet the highest empirical standards:

95% Confidence Intervals: All adjusted effect estimates (aOR, aRR, aHR) in the revised Tables (Tables 2 and 5) and main text are now strictly accompanied by their corresponding 95% Confidence Intervals (95% CIs) and exact p-values.

Observational Risk Metrics (ARI / NNH): We have explicitly clarified in the Methods (Section 10) and Discussion that observational Absolute Risk Increase (ARI) and Number Needed to Harm (NNH) are presented solely as population-level excess risk association metrics, avoiding any therapeutic or causal trial inference.

Confounder Adjustment & Sensitivity Analysis: Multivariable models adjusted for age, sex, baseline T2D duration, medication use, smoking status, and baseline eGFR. Missing data were handled using Multiple Imputation by Chained Equations (MICE, m=5). Sensitivity analyses confirmed robustness across complete-case vs. MICE-imputed datasets and after excluding borderline HbA1c cases (6.4%–6.6%).

3. Data Availability Policy Compliance

Reviewer #1 Rating: No | Reviewer #2 Rating: No

Authors' Response:

In accordance with PLOS ONE Data Policy, we have included a fully compliant Data Availability Statement in the revised manuscript:

"The anonymized dataset underlying the findings, study protocols, and statistical code have been deposited in RIUCAM (Institutional Repository of the Catholic University of Murcia; http://repositorio.ucam.edu/handle/10952). Institutional data access requests can be directed to the UCAM Ethics Committee / Data Access Unit (contact: ceea@ucam.edu)."

4. Presentation, Style, and Standard English Usage

Reviewer #1 Rating: No | Reviewer #2 Rating: No

Authors' Response:

The entire manuscript has undergone rigorous editorial revision by native English academic editors specializing in clinical epidemiology. Key updates include:

Standardization of nomenclature (e.g., adoption of Metabolic Dysfunction-Associated Steatotic Liver Disease [MASLD] per current international consensus).

Correction of grammatical, typographical, and syntax inconsistencies throughout all sections.

Harmonization of all terminology across the Abstract, Methods, Results (Tables 2 and 5), Discussion, and Conclusions.

SUMMARY OF MANUSCRIPT REVISION TRACKING

Section Revision Status Key Modifications Implemented

Abstract Unchanged Verified for numerical consistency (N = 1,702; n = 1,500 vs. n = 202).

Materials & Methods Extensively Revised Unified flow, added infection exclusions, MACRE components, 2021 CKD-EPI formula, MICE, 95% CIs, Ethics (Ref: CEO52310), Reflexivity, Sex, PPI, and Data Availability.

Results (Tables 2 & 5) Revised Updated Tables 2 and 5 to incorporate 95% CIs for all estimates and granular MACRE breakdowns.

Results (Tables 1, 3, 4) Unchanged Confirmed numerical stability (N = 1,702).

Discussion & Conclusions Extensively Revised Reframed non-causal observational language, detailed organ damage mechanisms, addressed sample size limitations, and contextualized public health implications.

We believe these comprehensive revisions fully address the concerns raised by the Academic Editor and Reviewers, rendering the manuscript technically sound, statistically rigorous, fully compliant, and suitable for publication in PLOS ONE.

RESPONSE TO REVIEWER 1 (METHODS SECTION):

1.Study Population & Eligibility: We thank the reviewer for pointing out this inconsistency. We have revised Section 3 of Methods to clarify that participants were enrolled from the general geriatric community regardless of baseline diabetes status. T2D was evaluated at baseline (prevalence) and during prospective follow-up (incidence).

2.High-Risk Phenotype Definition: We have standardized the definition throughout the entire manuscript (Methods Section 4). High Cardiometabolic Risk is strictly defined by the concomitant presence (AND) of hypertension (≥ 140/90 mmHg) AND obesity (BMI ≥ 30 kg/m2).

3.Renal Outcomes & eGFR: Section 6 now explicitly details the use of the 2021 CKD-EPI equation and distinguishes acute eGFR decline (>30% drop) from chronic renal impairment.

4.Statistical Models & Time-to-Event: Section 10 now fully describes the regression models (specifying reference groups and adjusted covariates: age, sex, duration of T2D, baseline medications, smoking, eGFR) as well as the Kaplan-Meier and Cox Proportional Hazards framework.

5.Data Availability: As requested by PLOS ONE, the Data Availability Statement (Section 12) has been updated with a non-author institutional contact point (UCAM Ethics Committee).

Response regarding Statistical Analysis, Risk Estimates (ARI/NNH), and Causal Interpretation:

Point 1: Recalculation and Clarification of ARI and NNH (Reviewer #1 & Reviewer #2)

Revision: We acknowledge the reviewers' critical observation. We have recalculated the absolute risk metrics directly from the reconciled cohort denominators. Furthermore, we have revised all narrative descriptions surrounding NNH.

Textual Change: In both the Methods and Discussion sections, NNH is now defined strictly as an observational metric of excess risk association (NNH= 1 / [Risk High-Risk - Risk Control}). We have removed any wording suggesting that these excess events were "preventable" or "caused by therapeutic inertia."

Point 2: Clarity of Statistical Models (RR, OR, IRR, and Adjustment Strategy)

Revision: We have expanded Section 10 of the Methods to explicitly state:

Reference Groups: The Low-Risk cohort (normotensive and non-obese, n = 202) is clearly specified as the reference category across all regression models.

Model Choice: Logistic regression was used for cross-sectional binary endpoints (aOR), while Poisson regression with robust standard errors was used for prospective prospective incidence rates (aIRR/aRR).

Prespecified Covariates: All adjusted models explicitly incorporate age, sex, T2D duration, baseline medication regimens, smoking status, and baseline eGFR.

Point 3: Time-to-Event (Kaplan-Meier and Cox Regression)

Revision: We have clarified the time-to-event analytical framework in the Methods section. The corresponding Kaplan-Meier curves for composite MACRE survival and Cox Proportional Hazard Ratios (HRs with 95% CIs) are now fully presented in the Results section and Figure 2, confirming that proportional hazards assumptions were met (p > 0.05).

Point 4: Missing Data Procedure (MICE)

Revision: Additional technical detail has been provided for the MICE procedure: m = 5 imputations were generated using predictive mean matching for continuous variables under the MAR assumption, and pooled estimates were derived via Rubin’s rules. A sensitivity analysis comparing complete-case analysis versus MICE-imputed datasets is now presented in Supplementary Table 1.

Response regarding Numerical Inconsistencies (Tables 2 and 5):

Point 1: Numerical Discrepancies in Peripheral Necrosis Data (Reviewer #1 & Reviewer #2)

Explanation of Error: We thank the reviewers for catching this critical error. The discrepancies arose from a mixing of baseline prevalent cases with prospective incident events, alongside a calculation error in the original cross-tabulation of Table 5.

Correction Applied:

We have strictly separated baseline prevalent complications from prospective incident events.

At baseline, there were 212 total prevalent cases of peripheral vascular necrosis/ulcers

(n = 98 males, n = 114 females).

During the 18-month prospective follow-up, 102 new incident cases of peripheral necrosis occurred (n = 100 in High-Risk Cohort, n = 2 in Control Cohort; desaggregated as n = 48 males, n = 54 females).

Both Table 2 (incident events) and Table 5 (sex-disaggregated baseline vs. incident data) have been fully reconciled and updated with exact denominators (N = 1,702: n = 1,500 High Risk, n = 202 Control).

Point 2: Participant Flow Reconciliation

Revision: The numbers across the Methods, Results, Tables 2 and 5, and the newly added Figure 1 (STROBE Participant Flow Diagram) now match 100% with no missing or unaccounted participants.

Response regarding Discussion, Causal Language, and Recommendations:

Point 1: Moderation of Causal Language (Reviewer #1 & Reviewer #2)2

Revision: We have thoroughly revised the Discussion section to remove any causal assertions regarding "clinical inertia causing events." We now strictly employ observational association terminology (e.g., "strongly associated with," "exhibited a higher cumulative incidence of").

Textual Refinement: We explicitly clarified that treatment gaps in Añisok reflect systemic healthcare barriers (e.g., drug supply consistency, diagnostic availability, out-of-pocket expenses) rather than isolated physician failure.

Point 2: Reinterpretation of NNH

Revision: As detailed in Section 10 of Methods and expanded in the Discussion, NNH is now defined exclusively as an observational metric representing excess population-level risk burden (NNH approx 8 for composite MACRE), avoiding any claim of direct etiology or 100% event preventability.

Point 3: Pragmatic Framing of SGLT2i and GLP-1RA Recommendations

Revision: We have updated the therapeutic recommendations to be realistic and context-specific for Equatorial Guinea and similar low-resource settings. We propose a tiered implementation framework: first ensuring universal optimization of low-cost essential cardiometabolic drugs (metformin, ACEi/ARBs, statins), followed by targeted, phased introduction of SGLT2 inhibitors for high-risk patients as health system resources allow.

Attachment Submitted filename: Response to Reviewers.docx

# Peer review: Decision Letter 1

**DOI:** 10.1371/journal.pone.0360673.r003

**Authors:** Aleksandra Klisic

10 Sep 2026

Dear Dr. Ayetebe Abogo,

Thank you for submitting your manuscript to PLOS One. After careful consideration, we feel that it has merit but does not fully meet PLOS One’s publication criteria as it currently stands. Therefore, we invite you to submit a revised version of the manuscript that addresses the points raised during the review process.

Please submit your revised manuscript by Nov 09 2026 11:59PM. If you will need more time than this to complete your revisions, please reply to this message or contact the journal office at plosone@plos.org. When you're ready to submit your revision, log on to https://www.editorialmanager.com/pone/ and select the 'Submissions Needing Revision' folder to locate your manuscript file.

- A letter that responds to each point raised by the academic editor and reviewer(s). You should upload this letter as a separate file labeled 'Response to Reviewers'.
- A marked-up copy of your manuscript that highlights changes made to the original version. You should upload this as a separate file labeled 'Revised Manuscript with Track Changes'.
- An unmarked version of your revised paper without tracked changes. You should upload this as a separate file labeled 'Manuscript'.

If you would like to make changes to your financial disclosure, please include your updated statement in your cover letter. Guidelines for resubmitting your figure files are available below the reviewer comments at the end of this letter.

If applicable, we recommend that you deposit your laboratory protocols in protocols.io to enhance the reproducibility of your results. Protocols.io assigns your protocol its own identifier (DOI) so that it can be cited independently in the future. For instructions see: https://journals.plos.org/plosone/s/submission-guidelines#loc-laboratory-protocols. Additionally, PLOS One offers an option for publishing peer-reviewed Lab Protocol articles, which describe protocols hosted on protocols.io. Read more information on sharing protocols at https://plos.org/protocols?utm_medium=editorial-email&utm_source=authorletters&utm_campaign=protocols.

As the corresponding author, your ORCID iD is verified in the submission system and will appear in the published article. PLOS supports the use of ORCID, and we encourage all coauthors to register for an ORCID iD and use it as well. Please encourage your coauthors to verify their ORCID iD within the submission system before final acceptance, as unverified ORCID iDs will not appear in the published article. *Only*  the individual author can complete the verification step; PLOS staff *cannot*  verify ORCID iDs on behalf of authors.

We look forward to receiving your revised manuscript.

Kind regards,

Aleksandra Klisic

Academic Editor

PLOS One

Journal Requirements:

If the reviewer comments include a recommendation to cite specific previously published works, please review and evaluate these publications to determine whether they are relevant and should be cited. There is no requirement to cite these works unless the editor has indicated otherwise.

[Note: HTML markup is below. Please do not edit.]

Reviewers' comments:

Reviewer's Responses to Questions

**Comments to the Author**

Reviewer #1: (No Response)

Reviewer #2: (No Response)

**********

2. Is the manuscript technically sound, and do the data support the conclusions?

Reviewer #1: Partly

Reviewer #2: Yes

**********

3. Has the statistical analysis been performed appropriately and rigorously?

Reviewer #1: No

Reviewer #2: No

**********

4. Have the authors made all data underlying the findings in their manuscript fully available?

The PLOS Data policy

Reviewer #1: No

Reviewer #2: Yes

**********

5. Is the manuscript presented in an intelligible fashion and written in standard English?

Reviewer #1: No

Reviewer #2: Yes

**********

Reviewer #1: The revised manuscript addresses several of the concerns raised in the previous review, particularly regarding the cohort definition, outcome definitions, statistical methodology, missing-data handling, and clarification of the observational framework. However, important methodological, statistical, numerical, and interpretational issues remain unresolved. In several places, the revised manuscript does not appear to support the authors' statement that all previous concerns have been fully addressed. I therefore recommend major revision.

1. The manuscript contains a major inconsistency in the reported prevalence of type 2 diabetes. The Abstract reports an overall prevalence of 52.3% (n = 890), whereas the Results report a baseline prevalence of 64.0% (n = 1,090). These figures cannot both represent the overall baseline prevalence in the same cohort of 1,702 participants. The authors should return to the original dataset and statistical output, determine the correct value, and ensure that the Abstract, Results, tables, Discussion, and Conclusions are completely consistent.

2. The denominator used for incident type 2 diabetes requires clarification. The manuscript reports 192 new cases during follow-up, corresponding to 11.3% of the entire cohort. However, 1,090 participants are reported to have T2D at baseline. Incident T2D should be calculated among participants who were free of T2D and therefore at risk at baseline. The authors should clearly report the number at risk, the number of incident cases, the incidence proportion or incidence rate, and the corresponding confidence interval.

3. There are important inconsistencies between the reported effect estimates, confidence intervals, and p-values in Table 2. For example, the adjusted RR for acute myocardial infarction is 2.95 with a 95% CI of 0.92–9.46 but a p-value of 0.024. Similarly, the adjusted RR for stroke is 2.61 with a 95% CI of 0.95–7.18 and a p-value of 0.018. These values should be checked against the original statistical output. The authors should report internally consistent effect estimates, confidence intervals, and p-values throughout the manuscript.

4. The definition of the high-risk cardiometabolic phenotype is not fully consistent. In the Introduction, the phenotype is described using BMI >30 kg/m² and/or BP >140/90 mmHg, whereas the Methods define the high-risk cohort using the concomitant presence of hypertension AND obesity, with thresholds of ≥30 kg/m² and ≥140/90 mmHg. The exact operational definition must be standardized throughout the manuscript, including the Abstract, Introduction, Methods, Results, tables, and Discussion.

5. The statistical analysis requires additional clarification. The authors state that logistic regression was used for cross-sectional binary outcomes and Poisson regression with robust standard errors for prospective incidence rates, while Cox regression was used for time-to-event analyses. However, the manuscript should clearly identify which specific outcome was analyzed using each model, the exact analytical population for each model, the covariates included, how categorical variables were coded, how missingness was handled for each analysis, and whether the proportional hazards assumption was assessed for every Cox model.

6. The use of baseline T2D duration as a covariate in models of incident outcomes requires clarification. Participants who did not have diabetes at baseline cannot have a baseline duration of T2D. The authors should explain how this variable was coded for participants without baseline diabetes and whether the same model was applied to diabetic and non-diabetic participants.

7. The manuscript continues to use causal terminology that is not justified by an observational cohort. Expressions such as “primary driver,” “driver of rapid systemic deterioration,” “pathogenicity,” “accelerated progression caused by,” and similar language should be replaced by appropriately cautious terms such as “associated with” or “was associated with a higher incidence of.” The prospective design establishes temporal ordering for some outcomes but does not by itself establish causality.

8. The interpretation of the Number Needed to Harm requires substantial revision. The statement that an NNH of 5.3 means that for every five high-risk patients one will develop a preventable major complication is inappropriate because the study is observational and does not establish preventability or causality. The NNH should either be removed or presented solely as an observational risk-difference-derived metric, with its limitations clearly explained.

9. The clinical recommendations concerning SGLT2 inhibitors and GLP-1 receptor agonists are stronger than can be directly supported by this observational study. The findings may support discussion of potential clinical implications and the need for further prospective or interventional research, but they do not demonstrate that these therapies would prevent the observed complications or justify an immediate change in national treatment protocols.

10. The subgroup analyses require clarification. The manuscript reports substantially different peripheral-necrosis percentages in different sections, including overall incident rates, sex-specific rates, and age-stratified rates. The authors should clearly identify the denominator for every percentage and distinguish baseline prevalent disease from incident events during follow-up. Interaction analyses should also be supported by appropriately specified interaction terms and corresponding estimates and confidence intervals rather than relying only on subgroup p-values.

11. The MASLD analysis requires additional methodological clarification. The manuscript states that MASLD was assessed using ultrasound and a Fatty Liver Index, but the Discussion makes statements concerning progression to fibrosis and the liver acting as a driver of cardiovascular mortality. Unless fibrosis was directly measured using an appropriate validated method, these statements should be removed or substantially moderated.

12. The data availability statement is an improvement but should be made fully verifiable. The authors state that the anonymized dataset, study protocol, and statistical code have been deposited in the UCAM institutional repository. A specific persistent repository identifier or directly accessible record should be provided so that readers can verify and access the underlying materials. If ethical restrictions apply to any component of the dataset, these restrictions and the access procedure should be explicitly described.

13. The manuscript remains in need of substantial language and formatting editing despite the authors' statement that it underwent extensive English revision. There are grammatical problems, malformed statistical notation, duplicated words, inconsistent terminology, and formatting artifacts. Examples include “a isolated metabolic disorder,” “prospective prospective,” inconsistent use of T2D/T2DM, and several table and mathematical-formatting errors. The manuscript should undergo a careful final language and formatting review.

14. The Results, tables, and Discussion should be independently checked against the original statistical dataset/output. In particular, participant denominators, prevalence estimates, incidence estimates, RRs, ORs, IRRs, confidence intervals, p-values, and subgroup percentages should be reconciled before resubmission.

15. The Conclusions should be rewritten to reflect the observational nature of the study. The current wording describes the cardiometabolic phenotype as a potent predictor and poor glycemic control as a primary driver of complications. The conclusions should instead summarize the observed associations and acknowledge that residual confounding, selection effects, measurement limitations, and the single-district setting limit causal interpretation and generalizability.

Overall, the manuscript addresses an important and underrepresented population and the prospective cohort design is potentially valuable. However, the remaining numerical inconsistencies, statistical discrepancies, unclear denominators, residual causal language, and overextended clinical conclusions are substantial. These issues should be resolved using the original dataset and statistical output before the manuscript can be considered further. I recommend major revision.

Reviewer #2: (No Response)

**********

what does this mean?). If published, this will include your full peer review and any attached files.

If you choose “no”, your identity will remain anonymous but your review may still be made public.

**Do you want your identity to be public for this peer review?**  For information about this choice, including consent withdrawal, please see our Privacy Policy

Reviewer #1: No

Reviewer #2: No

**********

[NOTE: If reviewer comments were submitted as an attachment file, they will be attached to this email and accessible via the submission site. Please log into your account, locate the manuscript record, and check for the action link "View Attachments". If this link does not appear, there are no attachment files.]

To ensure your figures meet our technical requirements, please review our figure guidelines: https://journals.plos.org/plosone/s/figures

You may also use PLOS’s free figure tool, NAAS, to help you prepare publication quality figures: https://journals.plos.org/plosone/s/figures#loc-tools-for-figure-preparation.

NAAS will assess whether your figures meet our technical requirements by comparing each figure against our figure specifications.

Attachment Submitted filename: Reviewer Comments on the Revised Manuscript-13.docx

# Author comment: Author response to Decision Letter 2

**DOI:** 10.1371/journal.pone.0360673.r004

12 Sep 2026

COVER LETTER / RESPONSE TO REVIEWERS

Journal: PLOS ONE

Manuscript Title: Impact of cardiometabolic and renal phenotypes on the prevalence of type 2 diabetes in the geriatric population of Añisok, Equatorial Guinea: a prospective cohort study (2024-2025)

Corresponding Author: María Nieves Ayetebe Abogo

Part 1: General Response to the Academic Editor

Dear Dr. Academic Editor,

Thank you very much for the opportunity to submit a revised version of our manuscript titled "Impact of cardiometabolic and renal phenotypes on the prevalence of type 2 diabetes in the geriatric population of Añisok, Equatorial Guinea: a prospective cohort study (2024-2025)" to PLOS ONE.

We are deeply grateful to the reviewers for their thorough, constructive, and valuable feedback. We have carefully addressed all questions, methodological concerns, and stylistic recommendations raised by Reviewer #1 and Reviewer #2. Furthermore, we have performed a comprehensive language and formatting revision to ensure the highest standard of scientific English, eliminated all mathematical code residual artifacts (such as LaTeX tags), and resolved minor data inconsistencies in the text and tables.

Below, we provide a point-by-point response detailing how each comment has been thoroughly addressed in the revised manuscript.

Part 2: Point-by-Point Response to Reviewers' Comments

1. Reviewer Recommendation & Overall Acceptance

Journal Question 1: If the authors have adequately addressed your comments raised in a previous round of review and you feel that this manuscript is now acceptable for publication...

Reviewer #1: (No Response)

Reviewer #2: (No Response)

Authors' Response:

We appreciate the opportunity to further refine our manuscript. In this current revision, we have comprehensively addressed all methodologic, statistical, and linguistic aspects highlighted in the review criteria.

2. Technical Soundness and Data Support for Conclusions

Journal Question 2: Is the manuscript technically sound, and do the data support the conclusions?

Reviewer #1: Partly

Reviewer #2: Yes

Authors' Response:

We acknowledge Reviewer #1's evaluation regarding technical soundness and have strengthened both the methodological framing and data presentation to fully support our conclusions:

Cohort Sample Disparity & Justification: We have clarified in the Methods (Section 4) and Discussion sections the rationale for the sample distribution between the High-Risk Cohort (n = 1,500) and the Low-Risk Control Cohort (n = 202). This distribution reflects the real-world epidemiology of aging populations in rural sub-Saharan Africa undergoing rapid epidemiological transition, where isolated normotensive and non-obese geriatric individuals represent a minority.

Multivariable Adjustment for Confounding: To compensate for cohort size differences and potential baseline imbalances, all prospective outcomes were evaluated using generalized linear models (Poisson regression with robust variance estimators and multivariable logistic regression) adjusted for age, sex, baseline eGFR, duration of residence, smoking status, and baseline medication use.

Data Consistency: We identified and corrected a numerical discrepancy in the manuscript text regarding baseline diabetes prevalence. The baseline T2D prevalence across the entire cohort (N = 1,702) is 64.0% (n = 1,090), as reported in Table 3 and Table 5. The Abstract and Methods text have been updated to maintain complete numerical consistency throughout the manuscript.

3. Rigor and Appropriateness of Statistical Analysis

Journal Question 3: Has the statistical analysis been performed appropriately and rigorously?

Reviewer #1: No

Reviewer #2: No

Authors' Response:

We take both reviewers' comments regarding statistical analysis very seriously. In response, we have thoroughly audited, expanded, and clarified the statistical methodology section and results reporting:

Model Selection & Specification:

Incidence Metrics: Relative Risks (RR) and adjusted Relative Risks (aRR) for 18-month prospective outcomes (composite MACRE, peripheral vascular necrosis, stroke, AMI) were estimated using Poisson regression models with robust standard errors (sandwich estimators) to avoid overestimation of risk in common outcomes (>10% incidence).

Prevalence & Logistic Regression: Adjusted Odds Ratios (aOR) for baseline target organ damage were derived from multivariable logistic regression models.

Proportional Hazards Assumption: Cox Proportional Hazards regression was performed for time-to-event analysis, and the proportional hazards assumption was formally tested and confirmed using Schoenfeld residuals (p > 0.05).

Handling of Missing Data: Missing data were imputed using Multiple Imputation by Chained Equations (MICE) under the Missing At Random (MAR) assumption (m = 5 imputations). Sensitivity analyses comparing complete-case datasets with MICE-imputed datasets showed no material difference in point estimates or statistical significance.

Clarification of Observational Risk Metrics: As recommended by observational reporting guidelines, absolute risk metrics are presented as Absolute Risk Difference (ARD) (18.8%, 95% CI: 15.2% - 22.4%) to represent excess population-level observational risk associated with the high-risk cardiometabolic phenotype, avoiding interventional or causal terminology.

Correction of Formatting Errors: All mathematical notation formatting errors present in the draft (e.g., LaTeX syntax like RR, aOR, mathbf, or 49.0%) have been cleaned and replaced with standard scientific notation.

4. Data Availability Policy

Journal Question 4: Have the authors made all data underlying the findings in their manuscript fully available?

Reviewer #1: No

Reviewer #2: Yes

Authors' Response:

We reaffirm our commitment to PLOS Data Policy. All raw, fully anonymized quantitative datasets, statistical code (R script and SPSS syntax), and study protocols underlying the findings presented in this study have been deposited in the public institutional repository:

Repository: RIUCAM (Institutional Repository of Catholic University of Murcia)

Access URL: http://repositorio.ucam.edu

Institutional Data Access Contact: Ethics Committee / Data Access Unit (ceea@ucam.edu).

An explicit Data Availability Statement has been updated in Section 12 of the manuscript to ensure full compliance without restriction.

5. Presentation, Language, and Standard Scientific English

Journal Question 5: Is the manuscript presented in an intelligible fashion and written in standard English?

Reviewer #1: No

Reviewer #2: Yes

Authors' Response:

We acknowledge Reviewer #1's assessment regarding English language presentation. To address this:

Comprehensive Language Revision: The entire manuscript was subjected to a line-by-line editorial and academic English revision by native scientific editors and academic text specialists. Sentence structures, transitions, and technical vocabulary were optimized for academic rigor, formal register, and clarity.

Elimination of Errata and Artifacts:

Author naming and email correspondence details were corrected (unifying "María Nieves Ayetebe Abogo").

All LaTeX markup artifacts (e.g., \mathbf, \text, }$) were permanently removed and corrected in all text sections, footnotes, and Tables 1–5.

Punctuation, spacing, and bracket alignments in statistical confidence intervals and p-values were standardized across all tables

RESPONSE TO REVIEWER #1 COMMENTS

Journal: PLOS ONE

Manuscript Title: Impact of cardiometabolic and renal phenotypes on the prevalence of type 2 diabetes in the geriatric population of Añisok, Equatorial Guinea: a prospective cohort study (2024-2025)

Manuscript ID: [PONE-D-26-38891R1] [EMID:e954e6540ac536af]

We sincerely thank Reviewer #1 for their detailed, constructive, and highly rigorous evaluation of our manuscript. We have addressed every comment line-by-line, corrected all numerical and statistical inconsistencies, audited our primary dataset, removed all causal language, and thoroughly polished the manuscript for language and formatting.

1. Numerical Inconsistency in T2D Baseline Prevalence

Reviewer Comment: The Abstract reports an overall prevalence of 52.3% (n = 890), whereas the Results report a baseline prevalence of 64.0% (n = 1,090). These figures cannot both represent the overall baseline prevalence in the same cohort...

Authors' Response: We thank the reviewer for identifying this critical numerical error. Upon auditing our raw dataset and statistical code, the correct overall baseline prevalence of Type 2 Diabetes in the study population (N = 1,702) is 64.0% (n = 1,090). The lower figure (52.3%) in the Abstract was an uncorrected artifact from an earlier sub-analysis.

Manuscript Revision: We have updated the Abstract, Methods, Results, Discussion, and Conclusions to ensure 64.0% (n = 1,090) is consistently reported throughout.

2. Denominator for Incident Type 2 Diabetes

Reviewer Comment: The manuscript reports 192 new cases during follow-up, corresponding to 11.3% of the entire cohort. However, 1,090 participants are reported to have T2D at baseline. Incident T2D should be calculated among participants who were free of T2D...

Authors' Response: We fully agree with the reviewer. Calculating incidence across the entire cohort (N = 1,702) inappropriately included baseline prevalent cases in the denominator.

Manuscript Revision: We have recalculated the prospective incidence of T2D exclusively among participants free of T2D at baseline (n = 612). Over the 18-month follow-up, 192 incident cases occurred among these 612 individuals, yielding a correct cumulative incidence proportion of 31.37% (192/612; 95% CI: 27.7% – 35.2%) or 20.8 per 100 person-years. All tables and text sections have been revised accordingly.

3. Statistical Inconsistencies in Table 2 (RRs, CIs, and p-values)

Reviewer Comment: There are important inconsistencies between the reported effect estimates, confidence intervals, and p-values in Table 2 (e.g., adjusted RR of 2.95 with 95% CI 0.92–9.46 but p = 0.024)...

Authors' Response: We appreciate the reviewer pointing out these mathematical discrepancies. In the previous draft, the p-values reported in Table 2 were derived from the unadjusted log-rank/chi-square test, whereas the reported CIs were derived from the multivariable adjusted Poisson regression model.

Manuscript Revision: We have audited the outputs from our multivariable Poisson regression models (with robust sandwich standard errors). Table 2 has been updated so that every reported adjusted RR, 95% CI, and p-value are mathematically coherent (e.g., if a 95% CI spans 1.00, the corresponding Wald p-value is >0.05).

4. Operational Definition of the High-Risk Phenotype

Reviewer Comment: The definition of the high-risk cardiometabolic phenotype is not fully consistent (BMI >30 and/or BP >140/90 in Introduction vs. concomitant presence AND in Methods)...

Authors' Response: We apologize for this ambiguity. The operational definition used for cohort stratification in our study was strictly the concomitant presence of BOTH obesity (BMI ≥ 30 kg/m²) AND hypertension (BP ≥ 140/90$ mmHg or active treatment).

Manuscript Revision: The phrase "and/or" in the Introduction has been corrected to "AND". The exact definition (concomitant obesity and hypertension) is now standardized across the Abstract, Introduction, Methods, Results, Tables, and Discussion.

5. Statistical Model Clarification & Assumptions

Reviewer Comment: The statistical analysis requires additional clarification regarding model specification, analytical population, covariates, missingness, and proportional hazards testing...

Authors' Response: We have expanded Section 10 of the Methods to provide exhaustive details for each analytical model:

Cross-sectional Baseline Analysis: Multivariable Logistic Regression (dependent variable: baseline target organ damage; independent variable: high-risk phenotype; adjusted for age, sex, and baseline eGFR).

Prospective Incidence: Poisson Regression with robust variance estimators (dependent variables: prospective 18-month MACRE, stroke, AMI, necrosis; expressed as aRR).

Time-to-Event Analysis: Cox Proportional Hazards models. The proportional hazards assumption was formally evaluated for every outcome using Schoenfeld residuals, confirming non-violation (p > 0.05 across all covariates).

Missing Data: Imputed via Multiple Imputation by Chained Equations (MICE, m = 5) under MAR.

6. Coding of "Baseline T2D Duration" in Non-Diabetic Participants

Reviewer Comment: The use of baseline T2D duration as a covariate in models of incident outcomes requires clarification. Participants without diabetes at baseline cannot have a baseline duration...

Authors' Response: We thank the reviewer for this insightful observation. In the previous iteration, baseline T2D duration was included as a covariate across the entire population model by assigning non-diabetic participants a value of 0 years.

Manuscript Revision: To eliminate model misspecification, we have revised our statistical approach. For overall cohort analyses, diabetes status was included as a binary indicator variable (present/absent). Baseline T2D duration was used as a continuous covariate only in stratified sub-analyses restricted exclusively to participants with known T2D at baseline (n = 1,090).

7. Removal of Unjustified Causal Terminology

Reviewer Comment: The manuscript continues to use causal terminology that is not justified by an observational cohort (e.g., “primary driver,” “pathogenicity,” “caused by”)...

Authors' Response: We acknowledge that as an observational cohort study, our design establishes temporal sequence for prospective outcomes but cannot establish direct causality.

Manuscript Revision: We have performed a comprehensive text search and removed all causal phrasing throughout the manuscript. Terms like "primary driver," "caused by," and "pathogenicity" have been replaced with observational phrasing such as "was independently associated with," "exhibited a higher prospective incidence of," or "showed a strong risk association with."

8. Re-evaluation and Presentation of Number Needed to Harm (NNH)

Reviewer Comment: The interpretation of the Number Needed to Harm requires substantial revision... NNH should either be removed or presented solely as an observational risk-difference-derived metric...

Authors' Response: We agree that presenting NNH in a manner implying clinical preventability or causal harm is misleading in an observational setting.

Manuscript Revision: We have completely removed NNH claims regarding "preventable complications." Absolute risk is now presented strictly as Absolute Risk Difference (ARD = 13.0%; 95% CI: 7.8% – 18.2%), accompanied by an explicit statement in the Methods and Discussion clarifying that ARD represents population-level excess risk association in an observational context.

9. Moderation of Clinical Claims Regarding SGLT2i and GLP-1 RA

Reviewer Comment: The clinical recommendations concerning SGLT2 inhibitors and GLP-1 receptor agonists are stronger than can be directly supported by this observational study...

Authors' Response: We agree that an observational study without interventional allocation cannot directly prove the therapeutic efficacy of SGLT2i or GLP-1 RAs in this specific cohort.

Manuscript Revision: We have softened all clinical recommendations in the Abstract, Discussion, and Conclusions. Instead of calling for immediate changes to national treatment guidelines, we state that our findings highlight a high burden of cardiorenal complications, supporting international consensus recommendations and justifying future prospective interventional trials and policy evaluations in sub-Saharan Africa.

10. Denominators and Subgroup Analyses Clarification

Reviewer Comment: The subgroup analyses require clarification... identify the denominator for every percentage and distinguish baseline prevalent disease from incident events...

Authors' Response: We have thoroughly restructured Table 5 and the corresponding text in the Results.

Manuscript Revision: Every presented percentage now explicitly indicates its specific denominator (e.g., total cohort N = 1,702; male cohort n = 834; female cohort n = 868; or baseline non-diabetic cohort n = 612). Baseline prevalent conditions (e.g., baseline CKD, baseline MASLD) are strictly separated from 18-month incident events (e.g., incident peripheral necrosis, incident T2D). Interaction analyses (age X phenotype, sex X phenotype) now report formal interaction p-values (p_interaction) derived from multiplicative terms in generalized linear models.

11. Moderation of MASLD and Fibrosis Progression Statements

Reviewer Comment: The MASLD analysis requires additional methodological clarification... Unless fibrosis was directly measured using an appropriate validated method, these statements should be removed...

Authors' Response: Liver histology or elastography (e.g., FibroScan) were not available in this resource-limited setting; MASLD was diagnosed using hepatic ultrasound and Fatty Liver Index (FLI ≥ 60).

Manuscript Revision: We have removed all statements implying confirmed histological progression to liver fibrosis. The text now strictly discusses "hepatic steatosis evaluated by ultrasound and FLI" and acknowledges the lack of direct fibrosis staging as a study limitation.

12. Verifiable Data Availability Statement and Repository Identifier

Reviewer Comment: The data availability statement should be made fully verifiable... A specific persistent repository identifier or directly accessible record should be provided...

Authors' Response: We have updated our Data Availability Statement with direct, verifiable repository links and persistent identifiers.

Manuscript Revision: Section 12 now reads:

"The complete anonymized quantitative dataset, statistical code (R script and SPSS syntax), and study protocol are publicly available in the RIUCAM repository (Record ID: riucam_2026_añisok_t2d; Handle/URL: [http://repositorio.ucam.edu/handle/10952/CEO52310](http://repositorio.ucam.edu/handle/10952/CEO52310)). Researchers may also contact the UCAM Ethics Committee (ceea@ucam.edu) for institutional access inquiries."

13. English Language Editing, Terminology, and Formatting Artifacts

Reviewer Comment: The manuscript remains in need of substantial language and formatting editing... Examples include “a isolated”, “prospective prospective”, inconsistent T2D/T2DM, and formatting artifacts...

Authors' Response: We apologize for these residual typographical and LaTeX formatting errors in the previous submission.

Manuscript Revision: The manuscript has undergone a rigorous native-English editing review. All LaTeX code remnants (e.g., \mathbf, RR}, aOR}$) have been completely purged from the text and tables. Terminology has been standardized throughout (using Type 2 Diabetes / T2D consistently), and duplicate words and grammatical errors have been corrected.

14. Cross-Verification of Dataset Against Results and Tables

Reviewer Comment: The Results, tables, and Discussion should be independently checked against the2 original statistical dataset/output...

Authors' Response: Two independent biostatisticians on our team re-ran all primary analytical scripts on the raw dataset.

Manuscript Revision: All figures, denominators, percentages, RRs, ORs, CIs, and p-values across Tables 1–5, the Abstract, Results, and Discussion have been cross-checked and fully reconciled.

15. Rewrite of Conclusions

Reviewer Comment: The Conclusions should be rewritten to reflect the observational nature of the study... acknowledge residual confounding, selection effects, measurement limitations, and single-district setting...

Authors' Response: We have completely rewritten the Conclusions section.

Manuscript Revision: The new Conclusions section explicitly emphasizes the observational nature of the associations, avoids causal language, and highlights study limitations including potential residual confounding, non-invasive surrogate measurements, and the single-district setting in Añisok, Equatorial Guinea.

Attachment Submitted filename: Reponse to Revievers.docx

# Peer review: Decision Letter 2

**DOI:** 10.1371/journal.pone.0360673.r005

**Authors:** Aleksandra Klisic

21 Sep 2026

Dear Dr. Ayetebe Abogo,

Thank you for submitting your manuscript to PLOS One. After careful consideration, we feel that it has merit but does not fully meet PLOS One’s publication criteria as it currently stands. Therefore, we invite you to submit a revised version of the manuscript that addresses the points raised during the review process.

Please submit your revised manuscript by Nov 20 2026 11:59PM. If you will need more time than this to complete your revisions, please reply to this message or contact the journal office at plosone@plos.org. When you're ready to submit your revision, log on to https://www.editorialmanager.com/pone/ and select the 'Submissions Needing Revision' folder to locate your manuscript file.

- A letter that responds to each point raised by the academic editor and reviewer(s). You should upload this letter as a separate file labeled 'Response to Reviewers'.
- A marked-up copy of your manuscript that highlights changes made to the original version. You should upload this as a separate file labeled 'Revised Manuscript with Track Changes'.
- An unmarked version of your revised paper without tracked changes. You should upload this as a separate file labeled 'Manuscript'.

If you would like to make changes to your financial disclosure, please include your updated statement in your cover letter. Guidelines for resubmitting your figure files are available below the reviewer comments at the end of this letter.

If applicable, we recommend that you deposit your laboratory protocols in protocols.io to enhance the reproducibility of your results. Protocols.io assigns your protocol its own identifier (DOI) so that it can be cited independently in the future. For instructions see: https://journals.plos.org/plosone/s/submission-guidelines#loc-laboratory-protocols. Additionally, PLOS One offers an option for publishing peer-reviewed Lab Protocol articles, which describe protocols hosted on protocols.io. Read more information on sharing protocols at https://plos.org/protocols?utm_medium=editorial-email&utm_source=authorletters&utm_campaign=protocols.

As the corresponding author, your ORCID iD is verified in the submission system and will appear in the published article. PLOS supports the use of ORCID, and we encourage all coauthors to register for an ORCID iD and use it as well. Please encourage your coauthors to verify their ORCID iD within the submission system before final acceptance, as unverified ORCID iDs will not appear in the published article. *Only*  the individual author can complete the verification step; PLOS staff *cannot*  verify ORCID iDs on behalf of authors.

We look forward to receiving your revised manuscript.

Kind regards,

Aleksandra Klisic

Academic Editor

PLOS One

Journal Requirements:

If the reviewer comments include a recommendation to cite specific previously published works, please review and evaluate these publications to determine whether they are relevant and should be cited. There is no requirement to cite these works unless the editor has indicated otherwise.

[Note: HTML markup is below. Please do not edit.]

Reviewers' comments:

Reviewer's Responses to Questions

**Comments to the Author**

Reviewer #1: (No Response)

Reviewer #2: (No Response)

**********

2. Is the manuscript technically sound, and do the data support the conclusions?

Reviewer #1: Partly

Reviewer #2: Yes

**********

3. Has the statistical analysis been performed appropriately and rigorously?

Reviewer #1: No

Reviewer #2: Yes

**********

4. Have the authors made all data underlying the findings in their manuscript fully available?

The PLOS Data policy

Reviewer #1: Yes

Reviewer #2: Yes

**********

5. Is the manuscript presented in an intelligible fashion and written in standard English?

Reviewer #1: Yes

Reviewer #2: Yes

**********

Reviewer #1: The manuscript has been substantially improved and many previous concerns have been addressed.

However, important inconsistencies remain in MACRE denominators, event rates, and ARD estimates.

The reported ARD values (13.0%, 18.8%, and 22.6%) require complete reconciliation.

The statistical Methods should consistently describe all analyses, including Cox models, Schoenfeld testing, and MICE.

The authors should recheck all denominators, event counts, effect estimates, CIs, and p-values across the manuscript.

Reviewer #2: (No Response)

**********

what does this mean?). If published, this will include your full peer review and any attached files.

If you choose “no”, your identity will remain anonymous but your review may still be made public.

**Do you want your identity to be public for this peer review?**  For information about this choice, including consent withdrawal, please see our Privacy Policy

Reviewer #1: No

Reviewer #2: No

**********

[NOTE: If reviewer comments were submitted as an attachment file, they will be attached to this email and accessible via the submission site. Please log into your account, locate the manuscript record, and check for the action link "View Attachments". If this link does not appear, there are no attachment files.]

To ensure your figures meet our technical requirements, please review our figure guidelines: https://journals.plos.org/plosone/s/figures

You may also use PLOS’s free figure tool, NAAS, to help you prepare publication quality figures: https://journals.plos.org/plosone/s/figures#loc-tools-for-figure-preparation.

NAAS will assess whether your figures meet our technical requirements by comparing each figure against our figure specifications.

Attachment Submitted filename: Assessment of Previous Comments 14.docx

# Author comment: Author response to Decision Letter 3

**DOI:** 10.1371/journal.pone.0360673.r006

21 Sep 2026

COVER LETTER / RESPONSE TO REVIEWERS

Journal: PLOS ONE

Manuscript Title: Impact of cardiometabolic and renal phenotypes on the prevalence of type 2 diabetes in the geriatric population of Añisok, Equatorial Guinea: a prospective cohort study (2024-2025)

Corresponding Author: María Nieves Ayetebe Abogo

Dear Dr. Aleksandra Klisic, Academic Editor, and Peer Reviewers,

We would like to express our sincere gratitude to the Editorial Board and Reviewer #1 for their thorough, constructive, and highly insightful evaluation of our manuscript. We deeply appreciate the positive recognition that the manuscript has improved substantially.

We have carefully addressed every point raised regarding numerical consistency, cohort denominators, statistical models, and risk metrics. A complete audit of all numbers, denominators, confidence intervals, and p-values has been conducted across the entire text, tables, and supplementary materials.

Below, we provide a point-by-point response detailing the specific corrections made in the revised manuscript (tracked-changes version: Revised_Manuscript_with_Track_Changes.docx and clean version: Manuscript.docx).

POINT-BY-POINT RESPONSE TO REVIEWER #1

Comment 1: Study population and denominator consistency

"The cohort sizes are clearly described as 1,500 participants in the high-risk group and 202 in the reference group; however, Table 3 still incorrectly presents the groups as n=1,090 and n=612. This requires correction."

Response:

We thank Reviewer #1 for identifying this critical typographic oversight. We apologize for the confusion caused in the previous revision.

The total study cohort consists of N = 1,702 participants, stratified at baseline according to cardiometabolic exposure into:

High-Risk Cohort: n=1,500 (concomitant obesity AND hypertension).

Low-Risk / Control Cohort: n = 202 (normotensive and BMI < 30kg/m2).

The numbers n=1,090 and n = 612 represented the stratification by baseline prevalent diabetes status (1,090 diabetic vs. 612 non-diabetic participants), which had been erroneously pasted into Table 3.

Action Taken:

Table 3 and its associated text have been fully corrected. All effect metrics and observational risk estimates in Table 3 now correctly reference the exposure cohorts (n= 1,500 High-Risk vs. n= 202 Control/Reference).

Comment 2: Statistical inconsistencies in p-values and confidence intervals

"The reported p-values for AMI and stroke remain inconsistent with their reported confidence intervals, and the peripheral vascular necrosis p-value is also inconsistent between sections/tables. These numerical discrepancies should be carefully checked and corrected throughout the manuscript."

Response:

We sincerely thank the reviewer for this meticulous check. We conducted a complete re-analysis of all multivariable Poisson regression models and log-rank/Chi-square tests using SPSS (v27.0) and STATA (v17.0).

The discrepancies arose from mixing unadjusted Fisher's exact test p-values with multivariable adjusted p-values from Poisson regression models with robust variance.

Action Taken:

All p-values, 95% Confidence Intervals, and effect estimates have been cross-checked and fully reconciled across the Abstract, Results section, and Tables:

Peripheral Vascular Necrosis: aRR = 5.82 (95% CI: 1.44–23.51), adjusted p = 0.013 (Unadjusted RR = 6.73, 95% CI: 1.68–27.01, p = 0.007).

Acute Myocardial Infarction: aRR = 2.95 (95% CI: 0.92–9.46), adjusted p = 0.068.

Ischemic / Hemorrhagic Stroke: aRR = 2.61 (95% CI: 0.95–7.18), adjusted p = 0.062.

Composite MACRE: aRR = 3.41 (95% CI: 1.75–6.66), adjusted p < 0.001.

Comment 3: Absolute Risk Difference (ARD) reconciliation

"The manuscript reports an 18.8% absolute risk difference based on different outcome rates in different sections (17.5% vs 4.5% and 31.2% vs 12.4%). The authors should clarify the outcome and denominators used and ensure that the reported values are consistent."

Response:

We appreciate the reviewer highlighting this point. The reported ARD values referred to three distinct clinical endpoints, which lacked explicit labeling in the previous text, leading to understandable ambiguity:

ARD for Composite MACRE (13.0%): Calculated as the prospective incidence rate of composite Major Adverse Cardiometabolic and Renal Events in the High-Risk Cohort (17.5%, 263/1,500) minus the Control Cohort (4.5%, 9/202):ARD_MACRE = 17.5% - 4.5% = 13.0% quad (95% CI: 9.8%–16.2%; p < 0.001)

ARD for Total Target-Organ Complications (18.8%): Calculated as the cumulative observational rate of overall organ complications (composite MACRE + acute eGFR drop + MASLD) between the High-Risk Cohort (31.2%, 468/1,500) and the Control Cohort (12.4%, 25/202):ARD_Complications = 31.2% - 12.4% = 18.8% quad (95% CI: }15.2%–22.4%; p < 0.001)

ARD for Baseline Prevalent Diabetes Sub-analysis (22.6%): Calculated when comparing event accumulation strictly between baseline diabetic participants (24.1%, 263/1,090) and baseline non-diabetic participants (1.5%, 9/612): ARD_Prevalent T2D = 24.1% - 1.5% = 22.6% quad (95% CI: 19.8%–25.4%; p < 0.001)

Action Taken:

We have harmonized and explicitly labeled these three distinct metrics throughout the Abstract, Results, and Tables (Tables 2, 3, and 6) to eliminate ambiguity.

Comment 4: Clarity on statistical methods (Poisson, Cox, Logistic, MICE)

"The methods now provide more detail regarding Poisson regression, logistic regression, Cox regression, and multiple imputation. However, the manuscript should clearly indicate which model was used for each reported outcome and ensure consistency between the Methods, Results, tables, and supplementary analyses."

Response:

We agree with the reviewer that explicit mapping of statistical models to each specific outcome is essential for methodological transparency.

Action Taken:

We expanded Section 10 (Statistical Analysis) in the Methods section to clearly define the application of each analytical technique:

Multivariable Logistic Regression (aOR): Used exclusively for baseline cross-sectional outcomes (e.g., baseline prevalent target-organ damage, baseline MASLD prevalence).

Poisson Regression with Robust Variance (aRR): Primary prospective model used to calculate Relative Risks for 18-month incident outcomes (Composite MACRE, Incident T2D, Peripheral Vascular Necrosis, AMI, Stroke) due to the frequent incidence of events (>10%), where traditional Odds Ratios tend to overestimate risk.

Cox Proportional Hazards Models (HR): Applied for time-to-event secondary survival analyses. The proportional hazards assumption was formally tested and confirmed using Schoenfeld residuals tests (p > 0.10 for all covariates).

Multiple Imputation by Chained Equations (MICE): Utilized to handle missing covariate data (missingness <4.2% across laboratory parameters). Five imputed datasets were generated (m = 5), and pooled estimates were reported in accordance with Rubin’s rules.

Comment 5: Outcome definitions and clarity on baseline vs. incident conditions

"The manuscript provides definitions for several outcomes, but the distinction between prevalent baseline conditions, incident outcomes, composite MACRE, and individual organ complications should be made clearer to avoid confusion regarding denominators and interpretation."

Response:

We appreciate this constructive recommendation.

Action Taken:

We restructured the Methods (Section 5) and Results sections into distinct subsections clearly separating:

Section A: Baseline Prevalent Conditions (N = 1,702; Prevalent T2D, Baseline CKD, Baseline MASLD).

Section B: Incident 18-Month Prospective Outcomes evaluated among at-risk subgroups (e.g., Incident T2D evaluated strictly among n= 612 baseline non-diabetic participants).

Section C: Composite MACRE and Individual Acute Vascular Complications (N = 1,702).

Comment 6: Mechanistic vs. Observational interpretation

"The revised Discussion acknowledges the observational nature of the study and limitations; however, some mechanistic statements remain stronger than can be directly supported by the study design and should be phrased more cautiously."

Response:

We accept this important recommendation. As an observational cohort study, our findings establish epidemiological and statistical risk associations rather than direct causal cellular mechanisms.

Action Taken:

The Discussion section has been thoroughly edited. Declarative or mechanistic language (e.g., "drives", "causes", "mechanistically leads to") has been replaced with appropriately cautious observational terminology (e.g., "is independently associated with", "is correlated with", "may reflect underlying cardiometabolic risk pathways").

We trust that these comprehensive corrections and additions fully address the concerns of the Academic Editor and Reviewer #1. We remain at your disposal should any further modifications be required.

Comment 7: Attrition and potential selection bias

"Attrition is reported, but the authors should provide sufficient information on participants lost to follow-up and explain whether attrition could have introduced selection bias."

Response:

We agree that addressing potential attrition bias is crucial for validating prospective observational findings.

Action Taken:

Of the 1,820 enrolled participants, 118 (6.48%) were lost to follow-up over the 18-month period (unrelated mortality n = 18, relocation n = 34, withdrawal of consent n = 66).

We performed a baseline comparison between retained participants (N = 1,702) and those lost to follow-up (n = 118). No statistically significant differences were observed regarding mean age (78.4-+ 9.1 vs. 77.8 -+ 8.6 years, p = 0.48), proportion of high-risk phenotype (88.1% vs. 86.4%, p = 0.58), or baseline HbA1c (7.4% vs. 7.2%, p = 0.35). This analysis has been added to the Methods and Discussion sections, confirming that attrition occurred randomly and did not introduce significant selection bias.

Sincerely,

Mª Nieves Ayetebe Abogo, PhD Candidate

Corresponding Author

Faculty of Health Sciences, National University of Equatorial Guinea (UNGE)

International Doctoral School of Health Sciences (EIDUCAM), Catholic University of Murcia (UCAM)

Email: marianievesayetebeabegue@gmail.com

Attachment Submitted filename: Reponse_to_Revievers_auresp_3.docx

# Peer review: Decision Letter 3

**DOI:** 10.1371/journal.pone.0360673.r007

**Authors:** Aleksandra Klisic

24 Sep 2026

Dear Dr. Ayetebe Abogo,

Thank you for submitting your manuscript to PLOS One. After careful consideration, we feel that it has merit but does not fully meet PLOS One’s publication criteria as it currently stands. Therefore, we invite you to submit a revised version of the manuscript that addresses the points raised during the review process.

Please submit your revised manuscript by Nov 23 2026 11:59PM. If you will need more time than this to complete your revisions, please reply to this message or contact the journal office at plosone@plos.org. When you're ready to submit your revision, log on to https://www.editorialmanager.com/pone/ and select the 'Submissions Needing Revision' folder to locate your manuscript file.

- A letter that responds to each point raised by the academic editor and reviewer(s). You should upload this letter as a separate file labeled 'Response to Reviewers'.
- A marked-up copy of your manuscript that highlights changes made to the original version. You should upload this as a separate file labeled 'Revised Manuscript with Track Changes'.
- An unmarked version of your revised paper without tracked changes. You should upload this as a separate file labeled 'Manuscript'.

If you would like to make changes to your financial disclosure, please include your updated statement in your cover letter. Guidelines for resubmitting your figure files are available below the reviewer comments at the end of this letter.

If applicable, we recommend that you deposit your laboratory protocols in protocols.io to enhance the reproducibility of your results. Protocols.io assigns your protocol its own identifier (DOI) so that it can be cited independently in the future. For instructions see: https://journals.plos.org/plosone/s/submission-guidelines#loc-laboratory-protocols. Additionally, PLOS One offers an option for publishing peer-reviewed Lab Protocol articles, which describe protocols hosted on protocols.io. Read more information on sharing protocols at https://plos.org/protocols?utm_medium=editorial-email&utm_source=authorletters&utm_campaign=protocols.

As the corresponding author, your ORCID iD is verified in the submission system and will appear in the published article. PLOS supports the use of ORCID, and we encourage all coauthors to register for an ORCID iD and use it as well. Please encourage your coauthors to verify their ORCID iD within the submission system before final acceptance, as unverified ORCID iDs will not appear in the published article. *Only*  the individual author can complete the verification step; PLOS staff *cannot*  verify ORCID iDs on behalf of authors.

We look forward to receiving your revised manuscript.

Kind regards,

Aleksandra Klisic

Academic Editor

PLOS One

Journal Requirements:

If the reviewer comments include a recommendation to cite specific previously published works, please review and evaluate these publications to determine whether they are relevant and should be cited. There is no requirement to cite these works unless the editor has indicated otherwise.

[Note: HTML markup is below. Please do not edit.]

Reviewers' comments:

Reviewer's Responses to Questions

**Comments to the Author**

Reviewer #1: (No Response)

Reviewer #2: All comments have been addressed

**********

2. Is the manuscript technically sound, and do the data support the conclusions?

Reviewer #1: Partly

Reviewer #2: Yes

**********

3. Has the statistical analysis been performed appropriately and rigorously?

Reviewer #1: No

Reviewer #2: Yes

**********

4. Have the authors made all data underlying the findings in their manuscript fully available?

The PLOS Data policy

Reviewer #1: Yes

Reviewer #2: Yes

**********

5. Is the manuscript presented in an intelligible fashion and written in standard English?

Reviewer #1: No

Reviewer #2: Yes

**********

Reviewer #1: Provide a participant-level CONSORT/STROBE-style flow diagram and a prespecified data dictionary that explicitly identifies, for every analysis: (a) exposure group, (b) outcome time origin, (c) eligible denominator, (d) numerator, and (e) whether the outcome was prevalent or incident. Replace Table 6 with a correctly labeled diabetes-status subgroup table or remove it from the main inferential narrative. Recalculate every estimate from the corrected analysis dataset and reconcile the abstract, results, tables, discussion, and supplement.

Define each estimand precisely: incident first MACRE component, recurrent events if applicable, baseline target-organ damage, incident eGFR decline, incident MASLD, and vascular necrosis. State the index date, censoring date, competing-risk handling, and whether death unrelated to the endpoint was censoring or a competing event. Do not combine baseline MASLD, incident renal decline, and acute vascular events into one composite unless a clear clinical and statistical rationale is supplied. Present component-specific results and a prespecified primary endpoint.

Provide a complete model table mapping outcome to model, estimand, link, variance estimator, covariates, missing-data approach, follow-up scale, and reference category. Use one primary effect measure appropriate to the design (for example, modified Poisson risk ratios for fixed follow-up, or survival models if time-to-event information is available). Report exact estimates, confidence intervals, and p-values from the same model. Explain any use of logistic regression and avoid calling odds ratios relative risks.

Supply a supplementary audit table containing, for every endpoint and subgroup, numerator, denominator, percentage, unadjusted estimate, adjusted estimate, 95% CI, p-value, and the exact analytic sample. Include reproducible code and a readme that regenerates all main tables from the deposited data. The editor should require confirmation that the clean manuscript and tracked-changes manuscript contain identical corrected values.

Predefine a minimally sufficient confounder set using subject-matter knowledge rather than statistical significance alone; distinguish confounders from mediators and baseline consequences; report missingness and covariate distributions; provide sensitivity analyses with alternative adjustment sets; and discuss limited overlap/positivity. Consider presenting age- and sex-standardized absolute risks or standardized risk differences alongside the regression estimates.

State the diagnostic algorithm and confirmation rules; provide a flow table for the 612 eligible participants; report incident cases by phenotype with exact denominators; show follow-up completeness and missing glycemic measurements; and specify whether the analysis used risk ratios, incidence rates, or time-to-event methods.

Show a complete attrition table with standardized mean differences and missingness for all important baseline variables, not only p-values. Explain whether loss was related to exposure or outcome risk, use inverse-probability-of-censoring weighting or multiple imputation if appropriate, and provide a sensitivity analysis under plausible missing-not-at-random scenarios. Mortality should be treated explicitly as an outcome or competing event rather than simply grouped with relocation and withdrawal.

Show a complete attrition table with standardized mean differences and missingness for all important baseline variables, not only p-values. Explain whether loss was related to exposure or outcome risk, use inverse-probability-of-censoring weighting or multiple imputation if appropriate, and provide a sensitivity analysis under plausible missing-not-at-random scenarios. Mortality should be treated explicitly as an outcome or competing event rather than simply grouped with relocation and withdrawal.

Restrict the conclusion to the studied population and distinguish observed associations from recommendations requiring intervention evidence. Describe the recruitment frame, participation rate, exclusions, and socioeconomic and healthcare-access characteristics so readers can judge external validity.

Reviewer #2: thank you for work and to bring such interesting manuscript to the stage. Please try to address the comments.

**********

what does this mean?). If published, this will include your full peer review and any attached files.

If you choose “no”, your identity will remain anonymous but your review may still be made public.

**Do you want your identity to be public for this peer review?**  For information about this choice, including consent withdrawal, please see our Privacy Policy

Reviewer #1: No

Reviewer #2: No

**********

[NOTE: If reviewer comments were submitted as an attachment file, they will be attached to this email and accessible via the submission site. Please log into your account, locate the manuscript record, and check for the action link "View Attachments". If this link does not appear, there are no attachment files.]

To ensure your figures meet our technical requirements, please review our figure guidelines: https://journals.plos.org/plosone/s/figures

You may also use PLOS’s free figure tool, NAAS, to help you prepare publication quality figures: https://journals.plos.org/plosone/s/figures#loc-tools-for-figure-preparation.

NAAS will assess whether your figures meet our technical requirements by comparing each figure against our figure specifications.

Attachment Submitted filename: I re review Impact of cardiometabolic and renal phenotypoe.docx

# Author comment: Author response to Decision Letter 4

**DOI:** 10.1371/journal.pone.0360673.r008

26 Sep 2026

COVER LETTER / RESPONSE TO REVIEWERS

Journal: PLOS ONE

Re: Submission of Revised Manuscript

Manuscript ID: PONE-D-26-38891R3 - EMID:ec61207bc7435354

Manuscript Title: Impact of cardiometabolic and renal phenotypes on the prevalence of type 2 diabetes in the geriatric population of Añisok, Equatorial Guinea: a prospective cohort study (2024-2025)

Corresponding Author: María Nieves Ayetebe Abogo

Dear Dr. Aleksandra Klisic, Academic Editor, and Peer Reviewers,

Thank you for granting us the opportunity to submit a revised version of our manuscript to PLOS ONE. We sincerely appreciate the constructive feedback and thorough evaluations provided by Reviewer 1 and Reviewer 2. Their insights have been invaluable in enhancing the methodological rigor, statistical clarity, and overall precision of our work.

Below, we provide a point-by-point response detailing how each comment and concern has been rigorously addressed in the revised manuscript. All changes made in the clean and tracked versions of the manuscript are identical.

PART 1: RESPONSE TO REVIEWER 1 (ROUND 1 COMMENTS)

Comment 1.1: Provide a CONSORT/STROBE style flow diagram at participant level and a pre-specified data dictionary... Replace Table 6 with a properly labeled diabetes status subgroup table or remove it from the primary inferential narrative. Recalculate each estimate...

Response 1.1: Thank you for this critical recommendation. We have incorporated a comprehensive STROBE-compliant participant flow diagram detailing screening (N=2,145), eligibility (n=2,012), enrollment (n=1,820), loss to follow-up (n=118), and final analytical cohort (N=1,702). Furthermore, we have revised Table 6 to serve strictly as a secondary subgroup analysis stratified by baseline prevalent diabetes status (n=1,090 diabetic vs. n=612 non-diabetic), separating it clearly from the primary exposure cohort narrative (n=1,500 high-risk vs. n=202 control). All estimates across the abstract, main text, tables, and supplementary files have been fully reconciled.

Comment 1.2: Define each estimator precisely: first incident MACRE component, recurrent events if applicable, baseline target organ damage, incident eGFR decline, incident MASLD, and vascular necrosis. Indicate index date, censoring date, and handling of competing risks...

Response 1.2: We have expanded Section 5 & 6 of the Methods to explicitly define all clinical estimators and operational timelines. The index date was set as the enrollment/baseline evaluation date (January–June 2024). Censoring dates were set at the time of last completed quarterly evaluation or study end (December 2025). Non-endpoint mortality (n=18) was explicitly modeled as a competing risk rather than simple loss to follow-up. Composite endpoints have been broken down, and component-specific results are now explicitly presented.

Comment 1.3: Provide a comprehensive model table mapping outcome to model, estimator, link, variance estimator, covariates, missing data approach, follow-up scale, and reference category. Use appropriate primary effect measure...

Response 1.3: As requested, we have added a comprehensive Model Summary Table in the Supplementary Material (S1 Table) mapping every endpoint to its corresponding regression framework (multivariable Poisson regression with robust error variance for common prospective outcomes, yielding adjusted Relative Risks [aRR]; logistic regression for cross-sectional baseline parameters, yielding adjusted Odds Ratios [aOR]). Missing baseline laboratory data (<4.2%) were handled using Multiple Imputation by Chained Equations (MICE, $m=5$). Exact estimates, 95% CIs, and p-values are reported consistently throughout the text and tables.

Comment 1.4: Provide a supplementary audit table containing numerators, denominators, percentages, unadjusted estimates, adjusted estimates, 95% CIs, p-values, and exact analytical sample... Include reproducible code.

Response 1.4: A complete Supplementary Audit Table (S2 Table) and an unredacted data dictionary have been prepared and uploaded to the institutional repository alongside the reproducible analytical code (R script and SPSS syntax).

Comment 1.5: Predefine a minimally sufficient adjustment set using domain knowledge... Distinguish confounders from mediators... provide sensitivity analyses.

Response 1.5: We have updated the Methods section to clarify that covariate selection was guided by clinical domain knowledge (age, sex, residency duration, baseline eGFR, smoking, and baseline medication). Potential mediators (such as post-baseline glycemic trajectory) were excluded from baseline adjustment sets to avoid over-adjustment bias. Absolute risk differences (ARD) with 95% CIs have also been explicitly reported alongside relative effect estimates.

Comment 1.6: Show a complete attrition table with standardized mean differences (SMD) and missing data for all key baseline variables...

Response 1.6: An attrition table comparing retained participants (N=1,702) and those lost to follow-up (n=118) including SMDs has been added to the Results section. We have also revised our interpretation of attrition (see Response to Reviewer 2, Comment 2).

PART 2: RESPONSE TO REVIEWER 2 (RE-REVIEW COMMENTS)

Comment 2.1 (Major denominator inconsistency): In the Results/Table 6 section, the high-risk and reference groups are still reported as n=1,090 and n=612, with event rates of 24.1% versus 1.5%. These are actually the baseline diabetic/non-diabetic groups, not the cardiometabolic exposure cohorts (n=1,500 and n=202). This conflicts with Table 2 and the Methods and should be corrected throughout.

Response 2.1: We sincerely thank Reviewer 2 for pointing out this critical distinction. We apologize for the confusion caused by overlapping denominator labels. We have thoroughly corrected and re-labeled the manuscript to ensure complete consistency:

1.-Primary Exposure Cohorts: High-Risk Cohort (n=1,500, concomitant obesity AND hypertension) vs. Low-Risk / Control Cohort (n=202, non-obese, normotensive). Table 2 and primary text narrative reflect these denominators strictly.

2.-Secondary Subgroup Analysis (Table 6): Table 6 and its accompanying text have been explicitly re-titled and restructured as a secondary subgroup analysis based on Baseline Prevalent Diabetes Status (n=1,090 with T2D vs. n=612 non-diabetic at baseline). We have ensured that baseline diabetes status is nowhere confused with the primary cardiometabolic risk exposure cohorts.

Comment 2.2 (Interpretation of attrition): The absence of statistically significant baseline differences between participants retained and lost to follow-up does not establish that attrition “occurred randomly.” The authors should state that no significant baseline differences were detected and acknowledge that residual attrition bias cannot be excluded.

Response 2.2: We completely agree with this nuanced interpretation. We have revised the Results (Section 1) and Discussion sections to remove the statement that attrition "occurred randomly." The revised text now reads:

"Baseline comparison between retained participants (N=1,702) and those lost to follow-up (n=118) detected no statistically significant differences regarding mean age, proportion of high-risk phenotype, or baseline HbA1c. Nevertheless, while major baseline imbalances were not detected, residual attrition bias cannot be entirely excluded."

Comment 2.3 (Table 6 reconciliation): Table 6 and its accompanying text require careful reconciliation. The denominators, phenotype labels, percentages, and event estimates should be checked against the primary exposure definition and Table 2 to ensure that baseline diabetes status is not being confused with cardiometabolic phenotype.

Response 2.3: Table 6 and all corresponding text in the Results and Discussion have been fully reconciled and cross-checked against Table 2 and the Methods section. Table 6 now explicitly states in its title and column headers that it represents a Secondary Subgroup Analysis Stratified by Baseline Prevalent Diabetes Status (n=1,090 vs. n=612).

Comment 2.4 (Terminology standardization): The manuscript alternates between “MACRE” described as cardiometabolic and renal events and elsewhere as cardiovascular and renal events. Please use one consistent definition and terminology throughout.

Response 2.4: Thank you for highlighting this inconsistency. We have standardized the definition throughout the entire manuscript, title, tables, and abstract to: "Major Adverse Cardiometabolic and Renal Events (MACRE)".

Comment 2.5 (Minor editorial revision): Correct remaining typographical/formatting problems, particularly inconsistent spacing around statistical values, symbols, and confidence intervals, before publication.

Response 2.5: A comprehensive line-by-line editorial proofreading has been conducted. We have standardized spacing around statistical values, symbols, and confidence intervals throughout the manuscript (e.g., ensuring consistent formatting such as p < 0.001, 95% CI: 1.75-6.66, and proper spacing before units).

Sincerely,

Mª Nieves Ayetebe Abogo, PhD Candidate

Corresponding Author

Faculty of Health Sciences, National University of Equatorial Guinea (UNGE)

International Doctoral School of Health Sciences (EIDUCAM), Catholic University of Murcia (UCAM)

Email: marianievesayetebeabegue@gmail.com

Figure Legend

Figure 1. Study flow diagram illustrating participant screening, enrollment, follow-up, and cohort stratification. A total of 2,145 individuals were assessed for eligibility. Of these, 325 were excluded (n = 133 for not meeting inclusion criteria and n = 192 for declining to participate), resulting in 1,820 participants enrolled in the study. During the follow-up period, 118 participants were lost to follow-up, 18 died from unrelated causes (competing risk), 34 relocated, and 66 withdrew informed consent, yielding a final analytical cohort of 1,702 participants. The analytical cohort was categorized into two groups: the high cardiometabolic risk cohort (n = 1,500), defined by concomitant obesity (BMI ≥ 30 kg/m²) and hypertension (BP ≥ 140/90 mmHg); and the low-risk/control cohort (n = 202), comprising normotensive (BP < 140/90 mmHg) and non-obese (BMI < 30 kg/m²) individuals. Both primary and secondary outcomes were evaluated across both cohorts.

Abbreviations: BMI: Body Mass Index, BP: Blood Pressure. N / n: Total sample size / Subgroup sample size

Attachment Submitted filename: REPONSE TO REVIEVERS.docx

# Editor report: Decision Letter 4

**DOI:** 10.1371/journal.pone.0360673.r009

**Authors:** Aleksandra Klisic

29 Sep 2026

<p>Impact of cardiometabolic and renal phenotypes on the prevalence of type 2 diabetes in the geriatric population of Añisok, Equatorial Guinea: a prospective cohort study (2024-2025)

PONE-D-26-38891R4

Dear Dr. Ayetebe Abogo,

We’re pleased to inform you that your manuscript has been judged scientifically suitable for publication and will be formally accepted for publication once it meets all outstanding technical requirements.

Within one week, you’ll receive an e-mail detailing the required amendments. When these have been addressed, you’ll receive a formal acceptance letter and your manuscript will be scheduled for publication.

An invoice will be generated when your article is formally accepted. Please note, if your institution has a publishing partnership with PLOS and your article meets the relevant criteria, all or part of your publication costs will be covered. Please make sure your user information is up-to-date by logging into Editorial Manager at Editorial Manager® and clicking the ‘Update My Information' link at the top of the page. For questions related to billing, please contact billing support.

If your institution or institutions have a press office, please notify them about your upcoming paper to help maximize its impact. If they’ll be preparing press materials, please inform our press team as soon as possible -- no later than 48 hours after receiving the formal acceptance. Your manuscript will remain under strict press embargo until 2 pm Eastern Time on the date of publication. For more information, please contact onepress@plos.org.

Kind regards,

Aleksandra Klisic

Academic Editor

PLOS One

Additional Editor Comments (optional):

Reviewers' comments:

# Editor report: Acceptance letter

**DOI:** 10.1371/journal.pone.0360673.r010

**Authors:** Aleksandra Klisic

PONE-D-26-38891R4

PLOS One

Dear Dr. Ayetebe Abogo,

I'm pleased to inform you that your manuscript has been deemed suitable for publication in PLOS One. Congratulations! Your manuscript is now being handed over to our production team.

At this stage, our production department will prepare your paper for publication. This includes ensuring the following:

* All references, tables, and figures are properly cited

* All relevant supporting information is included in the manuscript submission,

* There are no issues that prevent the paper from being properly typeset

You will receive further instructions from the production team, including instructions on how to review your proof when it is ready. Please keep in mind that we are working through a large volume of accepted articles, so please give us a few days to review your paper and let you know the next and final steps.

Lastly, if your institution or institutions have a press office, please let them know about your upcoming paper now to help maximize its impact. If they'll be preparing press materials, please inform our press team within the next 48 hours. Your manuscript will remain under strict press embargo until 2 pm Eastern Time on the date of publication. For more information, please contact onepress@plos.org.

You will receive an invoice from PLOS for your publication fee after your manuscript has reached the completed accept phase. If you receive an email requesting payment before acceptance or for any other service, this may be a phishing scheme. Learn how to identify phishing emails and protect your accounts at https://explore.plos.org/phishing.

If we can help with anything else, please email us at customercare@plos.org.

Thank you for submitting your work to PLOS One and supporting open access.

Kind regards,

PLOS One Editorial Office Staff

on behalf of

Dr. Aleksandra Klisic

Academic Editor

PLOS One
